# A Novel Role for the Longevity-Associated Protein SLC39A11 as a Manganese Transporter

**DOI:** 10.34133/research.0440

**Published:** 2024-08-07

**Authors:** Zhidan Xia, Biyao Tang, Xiaopeng Li, Xinran Li, Yangfan Jia, Jianwei Jiang, Jingyao Chen, Jingshu Song, Siyi Liu, Junxia Min, Fudi Wang

**Affiliations:** ^1^ The First Affiliated Hospital, The Second Affiliated Hospital, Institute of Translational Medicine, School of Public Health, Zhejiang Provincial Key Laboratory of Bioelectromagnetics, State Key Laboratory of Experimental Hematology, Zhejiang University School of Medicine, Hangzhou, China.; ^2^ School of Public Health, Basic Medical Sciences, The First Affiliated Hospital, Hengyang Medical School, University of South China, Hengyang, China.; ^3^ School of Public Health, School of Basic Medical Sciences, The First Affiliated Hospital, Xinxiang Medical University, Xinxiang, China; ^4^ The Core Facilities, Zhejiang University School of Medicine, Hangzhou, China.

## Abstract

The identification of aging- and longevity-associated genes is important for promoting healthy aging. By analyzing a large cohort of Chinese centenarians, we previously found that single-nucleotide polymorphisms (SNPs) in the *SLC39A11* gene (also known as *ZIP11*) are associated with longevity in males. However, the function of the SLC39A11 protein remains unclear. Here, we found that *SLC39A11* expression is significantly reduced in patients with Hutchinson–Gilford progeria syndrome (HGPS). In addition, we found that zebrafish with a mutation in *slc39a11* that significantly reduces its expression have an accelerated aging phenotype, including a shortened average lifespan, muscle atrophy and reduced swimming, impaired muscle regeneration, gut damage, and abnormal morphology in the reproductive system. Interestingly, these signs of premature aging were more pronounced in male zebrafish than in females. RNA-sequencing analysis revealed that cellular senescence may serve as a potential mechanism for driving this *slc39a11* deficiency-induced phenotype in mutant zebrafish. Moreover, immunofluorescence showed significantly increased DNA damage and reactive oxygen species signaling in *slc39a11* mutant zebrafish. Using inductively coupled plasma mass spectrometry (ICP-MS), we found that manganese significantly accumulates in *slc39a11* mutant zebrafish, as well as in the serum of both global *Slc39a11* knockout and hepatocyte-specific *Slc39a11* knockout mice, suggesting that this metal transporter regulates systemic manganese levels. Finally, using cultured human fibroblasts, we found that both knocking down *SLC39A11* and exposure to high extracellular manganese increased cellular senescence. These findings provide compelling evidence that SLC39A11 serves to protect against the aging process, at least in part by regulating cellular manganese homeostasis.

## Introduction

The essential trace element manganese (Mn) is a cofactor for activating a wide range of enzymes that play crucial roles in biological processes such as development, energy metabolism, immunomodulation, and defense against free radicals [1,2], and changes in Mn levels have been associated with a variety of diseases and pathophysiological conditions. For example, Mn deficiency can cause cognitive deficits and congenital disorders [3–5], while excess Mn can cause neurotoxicity and liver damage [6–8]. Thus, maintaining Mn homeostasis is essential for human health.

Mn homeostasis is mediated primarily by the metal transporters SLC39A8, SLC39A14, and SLC30A10 (also known as ZIP8, ZIP14, and ZnT10, respectively) [9]. SLC39A8 is located primarily at the apical surface of polarized hepatocytes, where it mediates the reuptake of Mn from bile into hepatocytes [10], while SLC39A14 and SLC30A10 are expressed primarily in hepatocytes and enterocytes, where they coordinately mediate Mn excretion into the bile and feces [11–16]. Mutations in these 3 transporters lead to inherited Mn transportopathies in humans [17]. Although these findings provide a mechanistic understanding of the role of these transporters in Mn homeostasis [18], an important question is whether other molecules are involved, and if so what role they play in the strict regulation of Mn homeostasis [19].

By performing a genome-wide association study (GWAS), we previously found that single-nucleotide polymorphisms (SNPs) in the *SLC39A11* (*ZIP11*) gene are significantly associated with longevity in male Chinese centenarians [20]. SLC39A11 was previously suggested to regulate zinc (Zn) levels when overexpressed in HEK293T cells exposed to extracellular Zn, suggesting that this protein may also serve as a Zn transporter [21]. Moreover, feeding wild-type (WT) mice a Zn-deficient diet leads to an up-regulation of Slc39a11 in the liver and colon [21,22]. In addition, SLC39A11 has been associated with the occurrence and/or development of several cancers, including bladder cancer, glioma, colorectal cancer, pancreatic cancer, and lung adenocarcinoma [23–27]. In HeLa cells, SLC39A11 was shown to be essential for regulating the cell cycle and cancer progression by maintaining nuclear Zn homeostasis [28]. However, the ion-selective properties and in vivo physiological function of SLC39A11 in vertebrates remain unknown. In addition, whether and how SLC39A11 affects lifespan, as well as the underlying mechanism, remain important open questions.

To characterize the in vivo physiological role of SLC39A11, we generated *slc39a11* mutant zebrafish, global *Slc39a11* knockout mice, and 2 tissue-specific *Slc39a11* knockout mice. The results obtained from these animal models provide compelling evidence that SLC39A11 functions as a conserved Mn transporter and plays an essential role in both aging and longevity.

## Results

### *slc39a11* mutant zebrafish have an accelerated aging phenotype

**Fig. 1.**
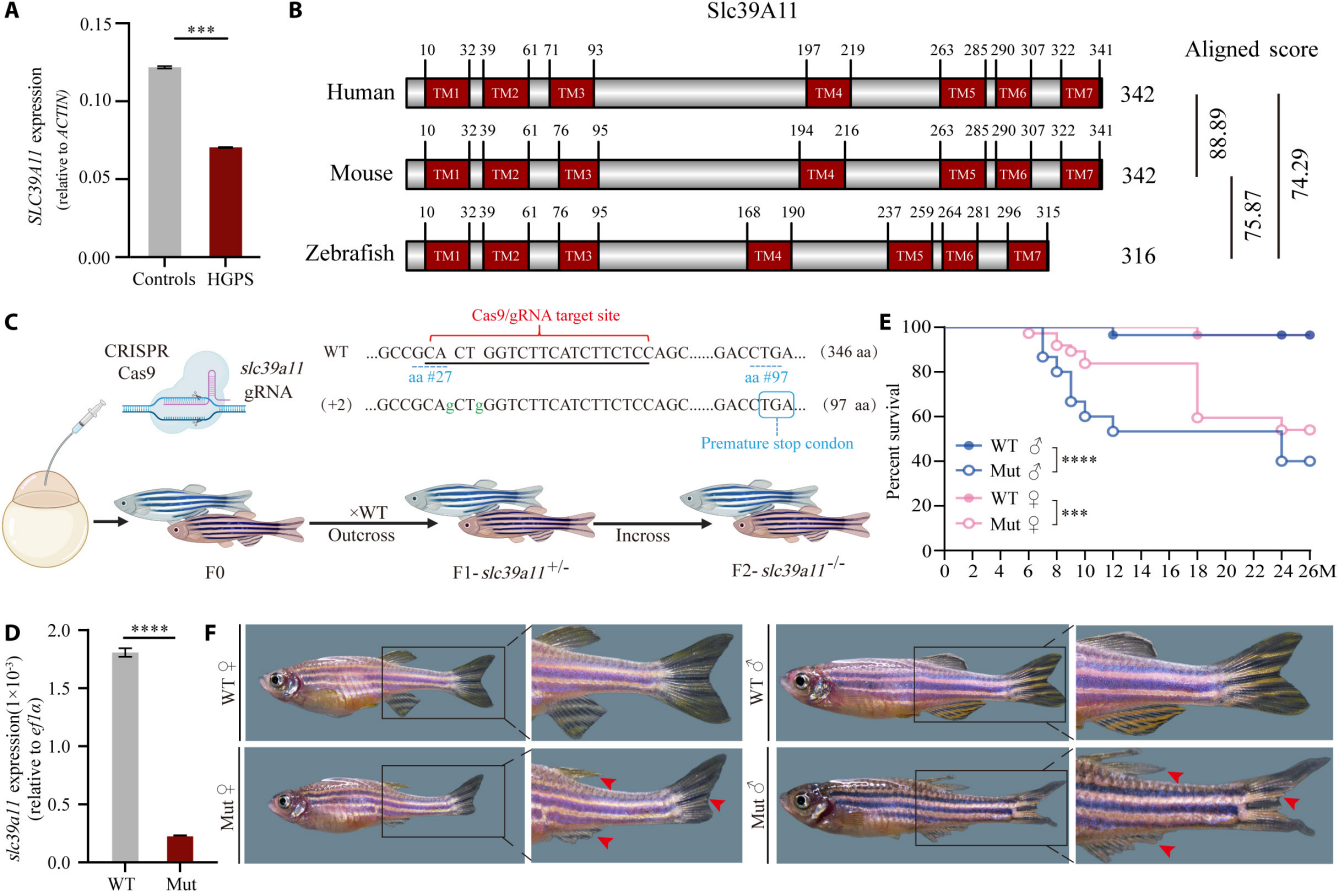
*slc39a11* mutant zebrafish have an accelerated aging phenotype. (A) Summary of *SLC39A11* mRNA measured in fibroblasts obtained from healthy controls and patients with Hutchinson–Gilford progeria syndrome (HGPS). (B) Alignment of the human, mouse, and zebrafish SLC39A11 proteins, with the 7 predicted transmembrane (TM) domains indicated; shown on the right are the homology scores obtained using ClustalW. (C) Strategy for generating *slc39a11* mutant zebrafish. (D) Summary of *slc39a11* mRNA measured in wild-type (WT) and *slc39a11* mutant (Mut) zebrafish embryos. (E) Survival curves for male and female WT and Mut zebrafish (*n* ≥ 20 per group). (F) Representative images of adult (12-month-old) zebrafish. The red arrowheads indicate damaged fins. ***P < 0.001 and ****P < 0.0001.

We first performed data mining for *SLC39A11* expression in human tissues by searching the Gene Expression Omnibus (GEO) Profiles database using the search term “SLC39A11 AND human[Organism] AND (aging OR age OR old OR senescence)”, which revealed one study involving human tissues obtained from patients with Hutchinson–Gilford progeria syndrome (HGPS), a rare fatal disease characterized by rapid, premature aging [29]. Specifically, we found significantly reduced *SLC39A11* expression in HGPS cells compared to healthy controls (Fig. 1A), which suggests that *SLC39A11* may be associated with aging. To investigate the physiological role of SLC39A11, we compared the protein sequences of human, mouse, and zebrafish orthologs (Fig. S1), revealing 88.89% similarity between the human and mouse orthologs and 74.29% similarity between the human and zebrafish orthologs (Fig. 1B). This relatively high degree of similarity suggests that SLC39A11 likely has a highly conserved function across these vertebrate species.

Next, we generated *slc39a11* mutant zebrafish using CRISPR/Cas9, in which 2 base pairs are inserted in the target site, causing a frame shift in the *slc39a11* mRNA (Fig. 1C). Using reverse transcription quantitative polymerase chain reaction (RT-qPCR), we confirmed that *slc39a11* mRNA levels are reduced by >90% in mutant animals compared to WT controls (Fig. 1D). Interestingly, *slc39a11* mutant zebrafish develop normally during embryogenesis and reach sexual maturity, but have a significantly lower survival rate starting at 8 months of age compared to WT controls, and male mutant zebrafish have a significantly lower survival rate compared to female mutants (Fig. 1E). In addition, both male and female mutant zebrafish have a less refractive epidermis and altered fin structures by approximately 12 months of age (Fig. 1F).

### Skeletal muscle tissues in mutant zebrafish have distinct aging-related characteristics

**Fig. 2.**
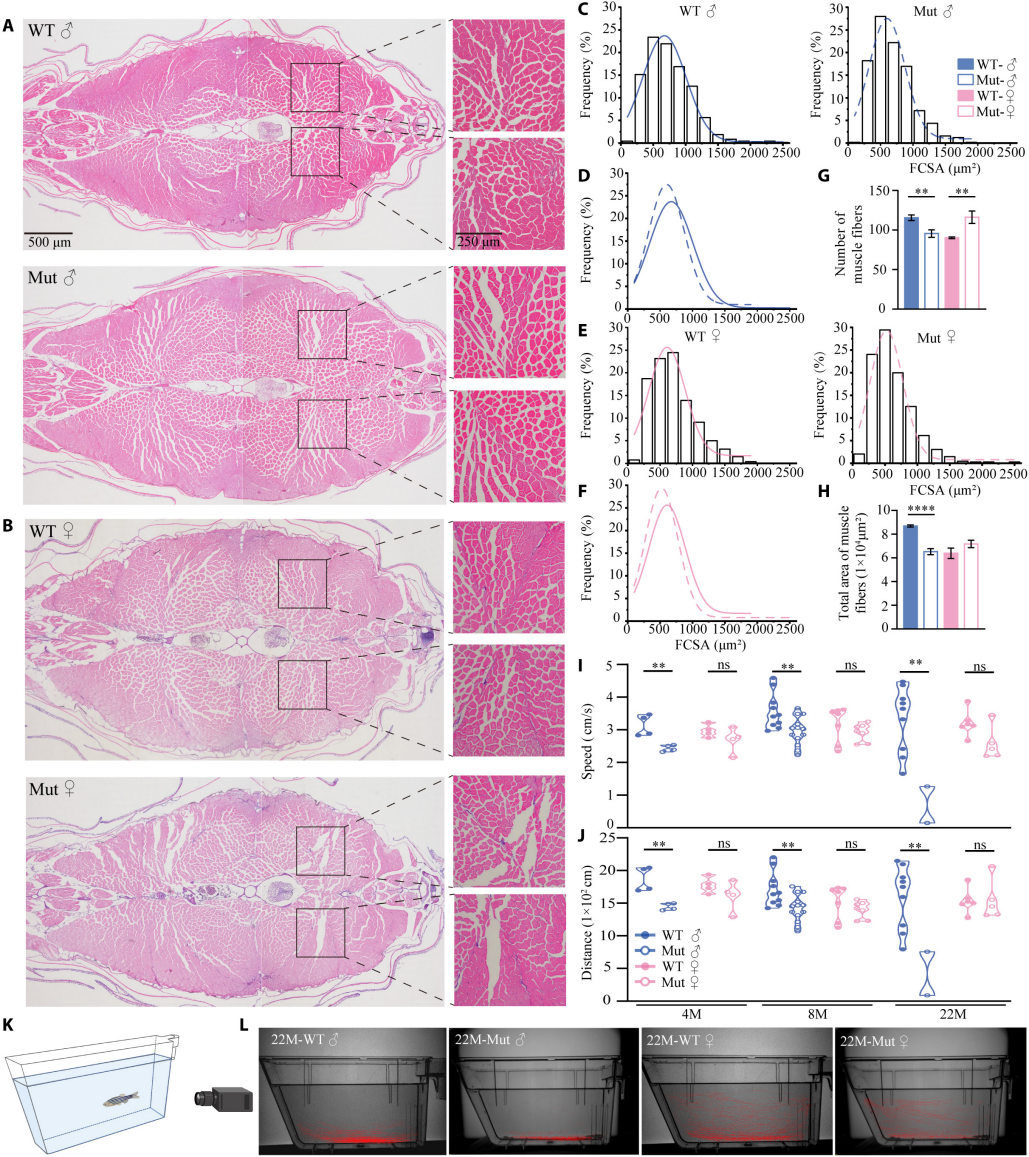
Skeletal muscle mass and locomotion are reduced in *slc39a11* Mut zebrafish. (A and B) H&E-stained muscle fibers in the tails of male (A) and female (B) WT and Mut zebrafish. (C to F) Gaussian distribution analysis of the area of individual muscle fibers in male (C and D) and female (E and F) zebrafish. Note that the curves shown in (D) and (E) are reproduced from (C) and (E), respectively, for comparison purposes only. (G and H) Summary of the total number (G) and total cross-sectional area (H) of muscle fibers in adult male and female WT and Mut zebrafish. (I and J) Summary of swimming speed (I) and distance traveled (J) by male and female WT and Mut zebrafish at 4, 8, and 22 months of age. (K) Diagram depicting the process for recording zebrafish swimming behavior (see Materials and Methods for details). (L) Representative images of each animal’s cumulative position in the tank tracked for 8 min, measured for 22-month-old male and female WT and Mut zebrafish. **P < 0.01, ***P < 0.001, ****P < 0.0001; ns, not significant.

Next, we stained muscle tissue sections with hematoxylin and eosin (H&E) and found thinner, less densely arranged muscle fibers in both male and female *slc39a11* mutants compared to controls (Fig. 2A and B). Examining the Gaussian distribution of muscle fiber cross-sectional area (FCSA) confirmed that the muscle fibers are thinner in both male and female *slc39a11* mutants (Fig. 2C to F). Moreover, the total number of muscle fibers in the male *slc39a11* mutants was lower compared to male controls (Fig. 2G), resulting in a decrease in total muscle fiber area (Fig. 2H). In contrast, the total number of muscle fibers was increased in the female *slc39a11* mutants (Fig. 2G), resulting in no difference in total muscle fiber area between female mutants and female controls (Fig. 2H). Thus, although both male and female *slc39a11* mutants develop muscle atrophy, only the male mutants have a net loss of muscle mass.

Consistent with these morphological changes in muscle tissues, starting at 4 months of age, male *slc39a11* mutants swim at a slower speed (Fig. 2I) and over a shorter distance (Fig. 2J), and this difference becomes progressively larger as the males reach 8 and 22 months of age. Moreover, we tracked the animals’ swimming patterns and found a clear difference between male mutants and WT males at 22 months of age (Fig. 1K and L). In contrast, we found no difference between female mutants and WT females with respect to swimming speed, distance, or pattern, even at 22 months of age (Fig. 2I to L).

**Fig. 3.**
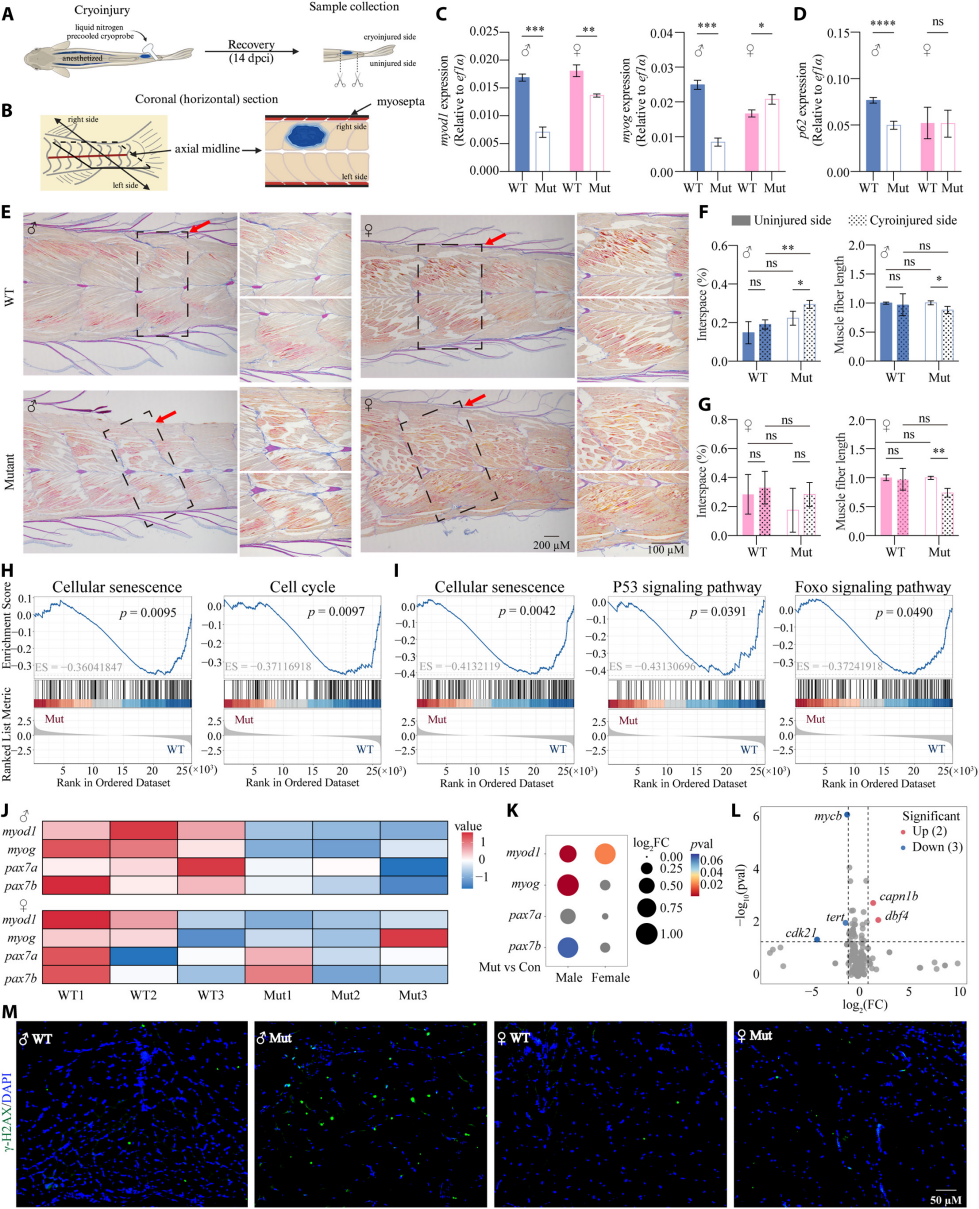
Impaired muscle regeneration and the potential aging mechanisms in the skeletal muscle of *slc39a11* Mut zebrafish. (A) Schematic diagram depicting the protocol for inducing unilateral cryoinjury. The cryoprobe was precooled in liquid nitrogen and immediately placed on the right side of the anesthetized zebrafish for 6 s; 14 days after cryoinjury (14 dpci), samples were collected and analyzed. (B) Schematic diagram depicting a coronal section through the caudal peduncle of a cryoinjured sample. (C and D) Summary of *myod1*, *myog*, and *p62* mRNA measured in the cryoinjured muscle tissue of male and female WT and Mut zebrafish. (E) Representative images showing the pattern of regenerated myomeres in male and female WT and Mut zebrafish. (F and G) Quantification of the gap (interspace) percentage (left panels) and relative length (right panels) of muscle fibers in the uninjured and cryoinjured sides in myomere samples obtained from male (F) and female (G) WT and Mut zebrafish. (H and I) Significant KEGG pathways related to the aging process in male (H) and female (I) groups that were enriched in Mut samples compared to WT samples, based on GSEA enrichment analysis. (J) Heatmap of the relative change in the expression of the indicated muscle regeneration-related marker genes in male and female WT and Mut samples. (K) Bubble chart analysis of the expression of the indicated muscle regeneration-related marker genes in male and female WT and Mut samples. (L) Volcano map of the expression of aging-related marker genes. (M) Fluorescence images of γ-H2AX immunostaining (green) in muscle fibers in male and female WT and Mut zebrafish; the nuclei were counterstained with 4′,6-diamidino-2-phenylindole (DAPI) (blue). *P < 0.05, **P < 0.01, ***P < 0.001, ****P < 0.0001.

Next, we subjected WT and mutant zebrafish to unilateral cryoinjury in order to induce profound damage to the musculature (Fig. 3A and B); we then evaluated the tissue’s regenerative capacity using RT-qPCR and AFOG (aniline blue, acid fuchsin, and orange G) histological staining [30]. The RT-qPCR results showed that 14 days after cryoinjury, the expression of the muscle regeneration markers *myod1* and *myog* was significantly decreased in male mutant muscles, and the expression of *myod1* was significantly decreased in female mutant muscles, compared to their respective WT tissues (Fig. 3C). In addition, expression of the wound clearance marker *p62* was also significantly decreased in male mutant muscles, but was unchanged in female mutant muscles (Fig. 3D). These data suggest that mutant males may have more severely impaired regenerative capacity than mutant females. Using AFOG histological staining, we found an obvious reduction in myomere regeneration in the cryodamaged side in *slc39a11* mutants, consistent with impaired regeneration, whereas virtually no damage in the myomeres remained in the cryodamaged side in WT controls, consistent with full regeneration (Fig. 3E). In addition, the regenerated muscle fibers in the mutant zebrafish were less densely packed and shorter compared to the fibers in the undamaged side, whereas the regenerated muscle fibers were similar to the undamaged fibers in the WT tissues (Fig. 3F and G). Together, these data indicate that the muscle fibers in *slc39a11* mutant zebrafish have an abnormal pattern and impaired regenerative capacity.

To examine the potential mechanisms underlying the aging-related phenotype in *slc39a11* mutant zebrafish, we performed RNA-sequencing (RNA-seq) analysis on muscle tissues obtained from WT and mutant zebrafish. Gene set enrichment analysis (GSEA) of the RNA-seq data revealed that aging-related pathways, including cellular senescence [Kyoto Encyclopedia of Genes and Genomes (KEGG): 04218] and cell cycle (KEGG: 04110), were significantly enriched in mutant male muscles compared to WT male samples (Fig. S2A and Fig. 3H), while cellular senescence (KEGG: 04218), the P53 signaling pathway (KEGG: 04115), and the Foxo signaling pathway (KEGG: 04068) were significantly enriched in mutant female muscles compared to WT female samples (Fig. S2B and Fig. 3I)[31,32]. Consistent with our qPCR results, heatmap analysis of the RNA-seq data showed that the expression levels of *myod1* and *myog* were significantly decreased in male mutant muscles, while the expression of *myod1* was significantly decreased in female mutant muscles (Fig. 3J). Overall, we found that the decreased expression of muscle regeneration markers was more pronounced in mutant males than in mutant females (Fig. 3K), suggesting that the molecular features that lead to impaired muscle regeneration are more pronounced in the mutant males than in the the mutant females. In addition, we found that expression of the telomerase reverse transcriptase (*tert*) was significantly decreased in male *slc39a11* mutants compared to WT males (Fig. 3L), suggesting reduced telomerase activity. Notably, using immunofluorescence, we also found significantly increased levels of γ-H2AX (phosphorylated histone H2AX, a biomarker of DNA damage) in male—but not female—*slc39a11* mutants compared to their respective WT controls (Fig. 3M), suggesting activation of DNA damage in the muscle tissues of male mutant zebrafish.

### *slc39a11* mutant zebrafish have altered visceral organ morphology

Given that aging is commonly associated with an abnormal “brain aging” phenotype [33], we next performed H&E staining of zebrafish brain sections and assessed neurological behaviors in order to determine whether *slc39a11* mutants develop this phenotype. However, histology revealed no apparent structural differences between *slc39a11* mutants and controls in the midbrain or hindbrain (Fig. S3A to D). In addition, an open-field test showed no difference in the duration of time in which the animals stayed in the central area of a circular tank, nor in the duration of time in which the animals stayed in the dark area of a light–dark tank (Fig. S2E to H). These findings suggest that the brain is not a primary organ affected in *slc39a11* mutant zebrafish.

**Fig. 4.**
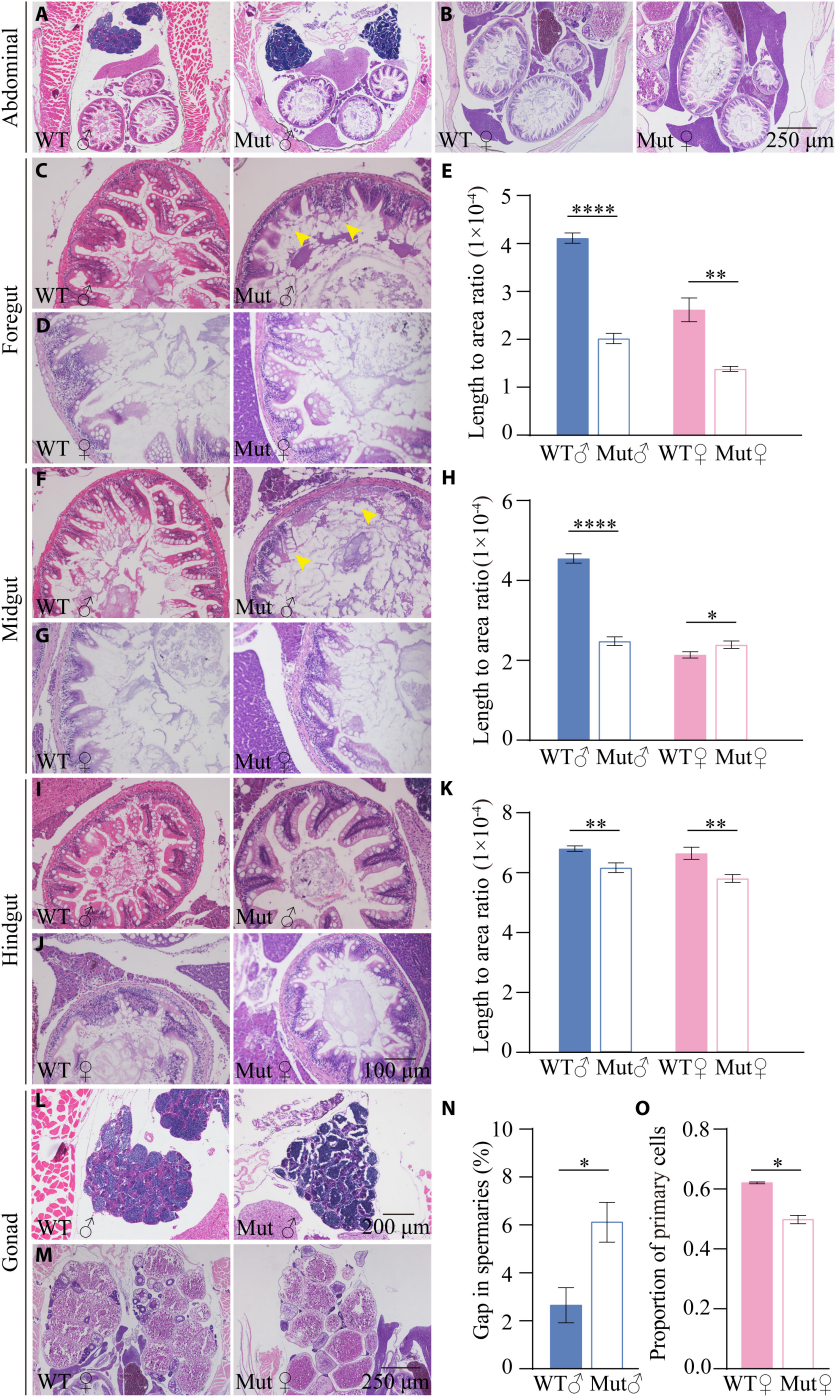
*slc39a11* Mut zebrafish have altered visceral organ and gonad morphology. (A and B) H&E-stained abdominal sections prepared from male (A) and female (B) WT and Mut zebrafish. (C to E) H&E-stained foregut sections prepared from male (C) and female (D) WT and Mut zebrafish, and summary of the ratio between villus length and foregut area (E). (F to H) H&E-stained midgut sections prepared from male (F) and female (G) WT and Mut zebrafish, and summary of the ratio between villus length and midgut area (H). (I to K) H&E-stained foregut sections prepared from male (I) and female (J) WT and Mut zebrafish, and summary of the ratio between villus length and foregut area (K). (L and M) H&E-stained spermary (L) and ovary (M) sections prepared from male (L) and female (M) WT and Mut zebrafish. (N and O) Summary of the percentage of gaps in the spermaries of male WT and Mut zebrafish (N), and summary of the percentage of primary oocytes in the ovaries of female WT and Mut zebrafish (O). *P < 0.05, **P < 0.01, and ****P < 0.0001.

Next, we expanded our histological analysis to visceral organs. We found that male *slc39a11* mutants have both fewer and more damaged villi in the foregut and midgut (Fig. 4A to J); moreover, the villi were significantly shorter in both the foregut and midgut of male mutants compared to controls (Fig. 4E and H). In contrast, the villi in the foregut—but not in the midgut—were shorter in the female mutants compared to controls (Fig. 4E and H). These morphological changes were considerably less pronounced—but still significant—in the hindgut of both male and female *slc39a11* mutants compared to their respective controls (Fig. 4I to K).

We also found differences between *slc39a11* mutants and controls with respect to reproductive organs. Specifically, the gonads contained less densely packed spermaries in the male *slc39a11* mutants compared to controls (Fig. 4L and N), while the ovaries contained a lower percentage of primary oocytes in the female mutants compared to controls (Fig. 4M and O). Given the previous report that the *klotho* gene, which encodes the anti-aging protein Klotho, is highly expressed in zebrafish testis and ovaries [34], we measured *klotho* mRNA levels in these tissues and found significantly decreased expression in the testis and ovaries of male and female mutants, respectively, compared to their respective controls (Fig. S3I). Together, these results indicate that the decrease in *slc39a11* expression in the mutant zebrafish causes an aging phenotype that is more severe in males than females.

### SLC39A11 functions as a systemic Mn transporter in vertebrates

**Fig. 5.**
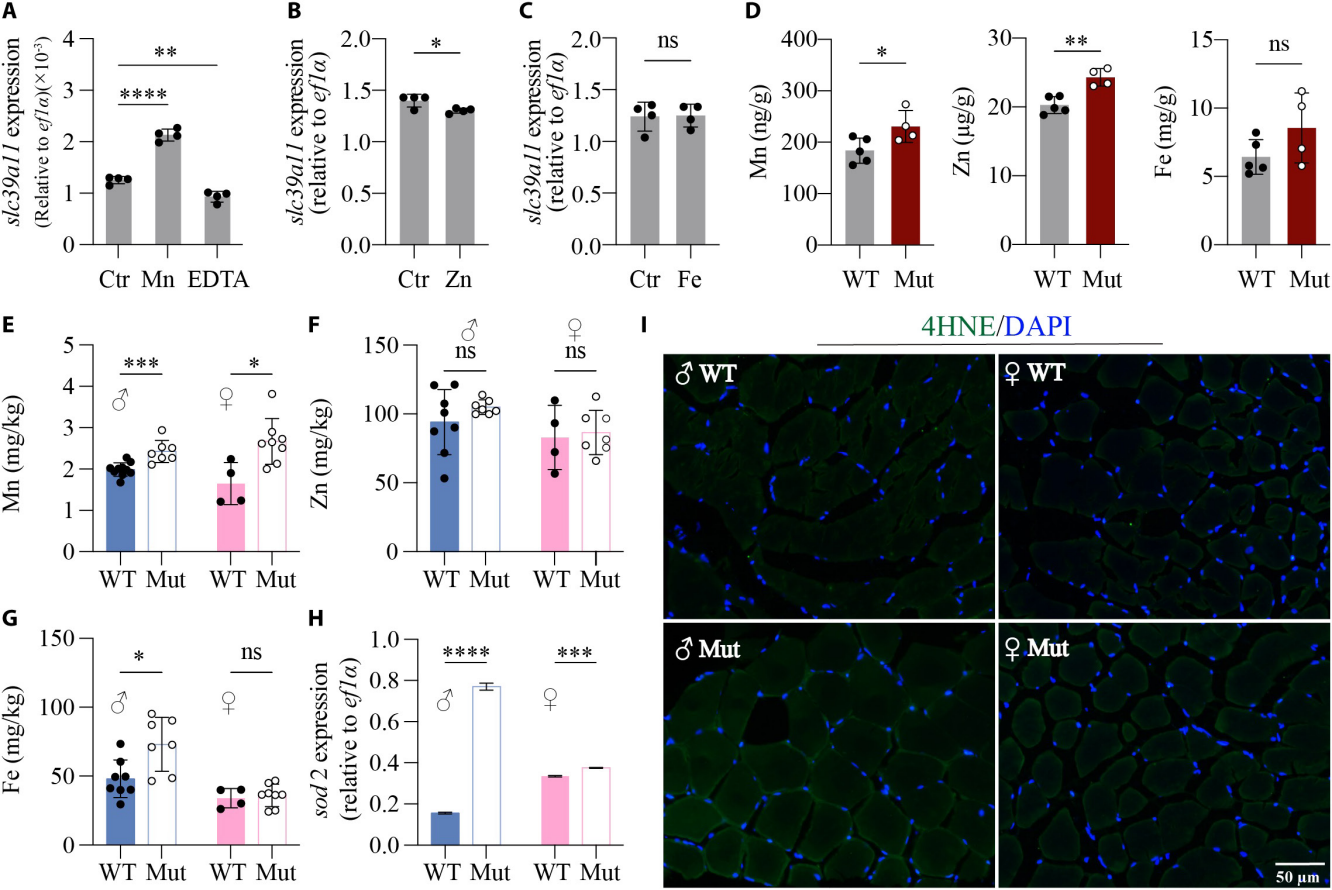
Mn exposure increases *slc39a11* expression in zebrafish, and *slc39a11* Mut zebrafish have increased Mn content. (A to C) Summary of *slc39a11* mRNA measured in untreated (Ctr) WT zebrafish embryos and embryos treated with either 100 μM MnCl_2_ or the chelator EDTA (A), 100 μM ZnSO_4_ (B), or 100 μM ferric citrate (C). (D) Summary of Mn, Zn, and Fe content measured in WT and *slc39a11* Mut zebrafish embryos. (E to G) Summary of Mn (E), Zn (F), and Fe (G) content measured in male and female WT and Mut zebrafish adults. (H) Summary of *sod2* mRNA measured in the muscle tissue of male and female WT and Mut zebrafish. (I) Fluorescence images of 4-HNE immunostaining (green) in muscle fibers of male and female WT and Mut zebrafish; the nuclei were counterstained with DAPI (blue). *P < 0.05, **P < 0.01, ***P < 0.001, ****P < 0.0001.

To determine the effect of various cations on *slc39a11* expression, WT zebrafish embryos were exposed to a solution containing Mn^2+^, Zn^2+^, or Fe^3+^. We found that Mn^2+^ significantly increased *slc39a11* expression, whereas treating embryos with the chelator EDTA significantly decreased *slc39a11* expression (Fig. 5A); in contrast, Zn^2+^ slightly—but significantly—decreased *slc39a11* expression (Fig. 5B), and Fe^3+^ had no effect (Fig. 5C). Thus, *slc39a11* expression is sensitive to changes in Mn^2+^ levels. We then used inductively coupled plasma mass spectrometry (ICP-MS) to measure Mn, Zn, and Fe levels and found significantly higher levels of Mn and Zn in mutant embryos compared to WT controls, but no significant difference in Fe levels (Fig. 5D). In adults, we also found significantly higher levels of Mn in both male and female mutants compared to their respective WT controls (Fig. 5E), as well as significantly higher Fe levels in male mutants (Fig. 5G); in contrast, we found no difference in Zn levels (Fig. 5F), and no difference in Fe levels in female mutants (Fig. 5G). These results suggest that while Mn affects *slc39a11* expression, the Slc39a11 protein also regulates Mn levels.

Oxidative stress serves as a mechanism common to aging and Mn toxicity [35]. We therefore measured *sod2* mRNA (which encodes the antioxidant enzyme superoxide dismutase) and production of the reactive oxygen species (ROS) metabolite 4-hydroxynonenal (4-HNE) in the muscle tissues of *slc39a11* mutants and WT controls. We found significantly increased *sod2* expression in both male and female mutants (Fig. 5H), as well as increased 4-HNE in *slc39a11* mutant muscle fibers compared to WT controls (Fig. 5I), and both effects were more pronounced in the male mutants than in the female mutants. Based on these results, we hypothesize that Mn-induced oxidative stress may serve as a possible mechanism in the aging phenotype observed in *slc39a11* mutant zebrafish.

**Fig. 6.**
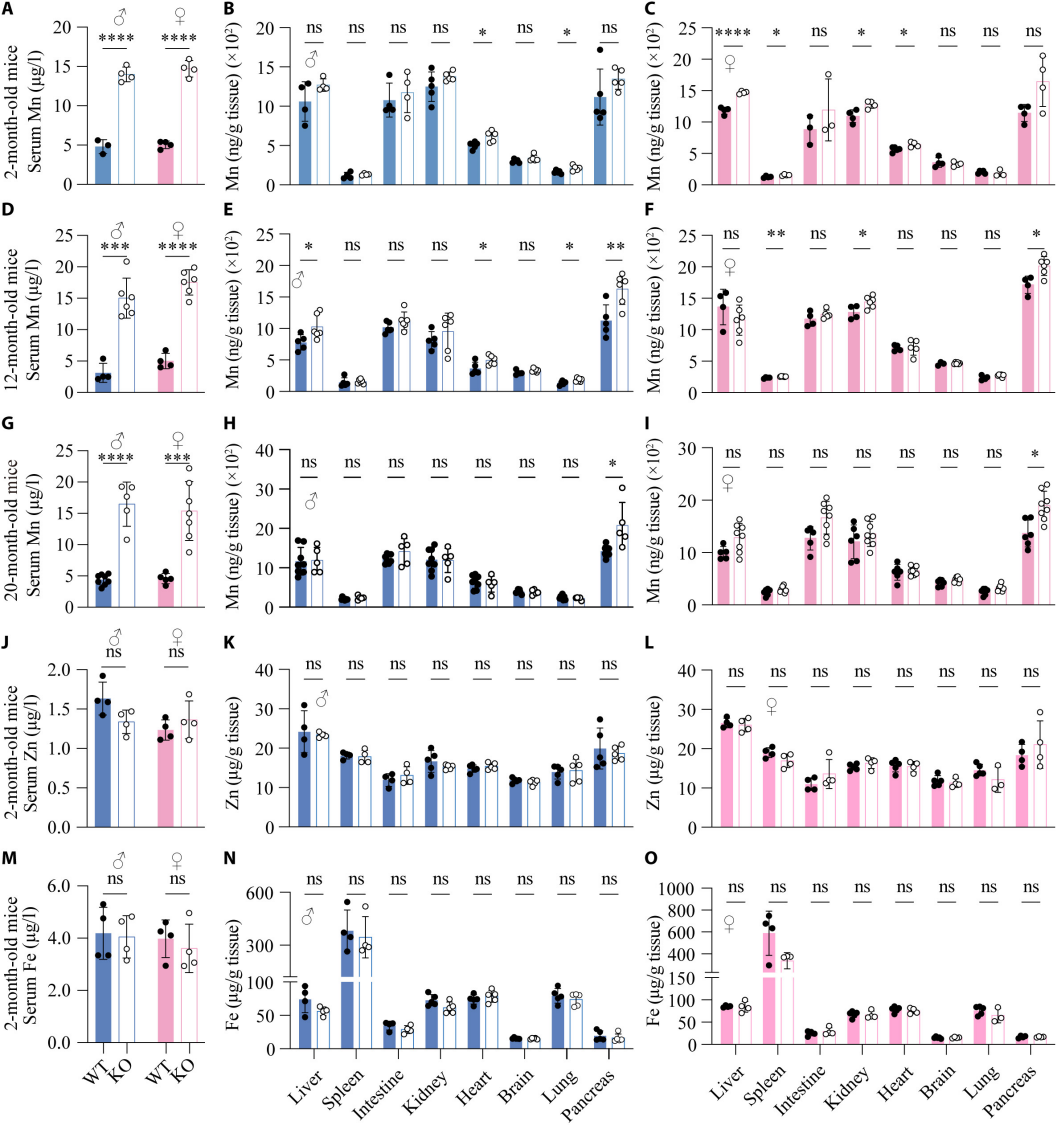
Serum Mn levels are elevated in global *Slc39a11* knockout mice. (A to I) Mn levels in the serum and the indicated tissues were measured in 2-month-old (A to C), 12-month-old (D to F), and 20-month-old (G to I) male and female WT and *Slc39a11* knockout (KO) mice. (J to O) Summary of Zn (J to L) and Fe (M to O) content measured in the serum and indicated tissues of 2-month-old male and female WT and KO. *P < 0.05, **P < 0.01, ***P < 0.001, ****P < 0.0001.

To examine whether SLC39A11 is functionally conserved in mammals, we generated global *Slc39a11* knockout (*Slc39a11*^−/−^) mice (Fig. S4A); loss of *Slc39a11* expression was confirmed in all tissues tested in both male (Fig. S4B) and female (Fig. S4C) mice using RT-qPCR. We found that *Slc39a11*^−/−^ mice develop normally and do not exhibit any abnormal neurobehavioral properties (Fig. S5). We then measured Mn concentrations in serum and various tissues at 2, 12, and 20 months of age using ICP-MS (Fig. 6). We found that at all 3 ages, serum Mn concentrations were significantly higher in both male and female *Slc39a11*^−/−^ mice (Fig. 6A, D, and G), as well as in certain tissues in both sexes at 2 (Fig. 6B and C), 12 (Fig. 6E and F), and 20 (Fig. 6H and I) months of age, compared to their respective controls. In contrast, we found no difference between *Slc39a11*^−/−^ and control mice with respect to serum or tissue levels of Zn (Fig. 6J to L), Fe (Fig. 6M to O), Mg (Fig. S6A), Ca (Fig. S6B), or Cu (Fig. S6C). Taken together, both our zebrafish and mouse data indicate that a loss of SLC39A11 expression leads to systemic Mn accumulation, suggesting that SLC39A11 plays a key role in regulating Mn homeostasis under physiological conditions and functions as a conserved Mn transporter in vertebrates.

### SLC39A11 regulates Mn homeostasis in the liver and intestine

**Fig. 7.**
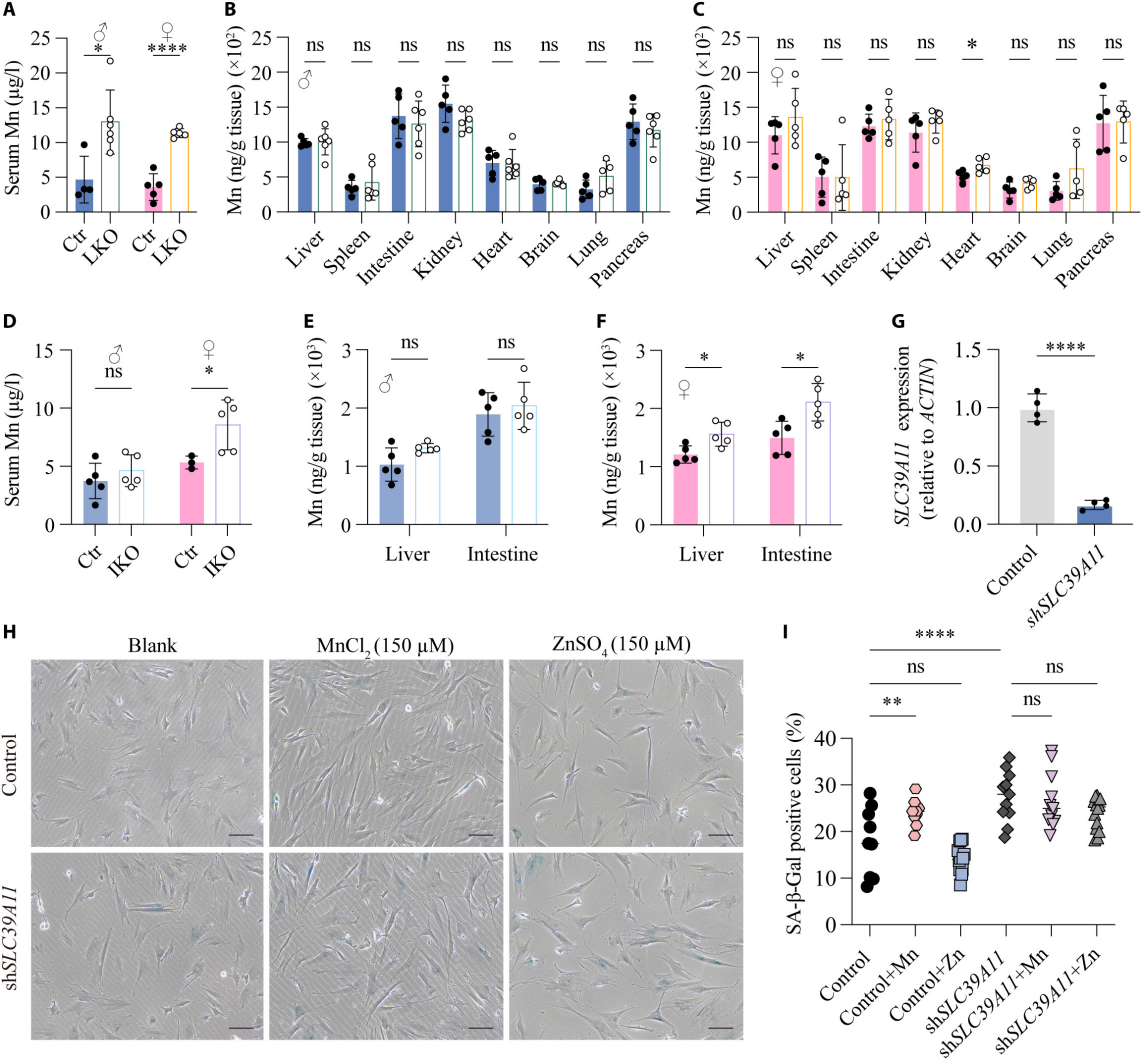
Tissue-specific *Slc39a11* knockout mice have increased serum Mn levels, and knocking down *SLC39A11* in vitro increases cellular senescence. (A to C) Summary of Mn levels measured in the serum (A) and indicated tissues (B and C) in 2-month-old *Slc39a11^flox/flox^* (Ctr) and liver-specific *Slc39a11* knockout (LKO) male (B) and female (C) mice. (D to F) Summary of Mn levels measured in the serum (D) and liver and intestine (E and F) of 2-month-old *Slc39a11^flox/flox^* (Ctr) and intestine-specific *Slc39a11* knockout (IKO) male (E) and female (F) mice. (G) Summary of *SLC39A11* mRNA measured in control fibroblasts and fibroblasts expressing an shRNA against *SLC39A11* (sh*SLC39A11*). (H) Representative phase contrast images of control fibroblasts and fibroblasts expressing sh*SLC39A11* stained for SA-β-Gal after exposure to no additional metal ions (blank), 150 μm MnCl_2_, or 150 μM ZnSO_4_. Scar bar, 10 µm. (I) Summary of the percentage of SA-β-Gal-positive cells treated as indicated. *P < 0.05, **P < 0.01, ****P < 0.0001.

Given that the liver and small intestine play a role in Mn distribution and metabolism [36], we generated liver-specific *Slc39a11* knockout (LKO) and intestine-specific *Slc39a11* knockout (IKO) mice by crossing conditional *Slc39a11* knockout mice with Alb-Cre and Villin-Cre mice, respectively; loss of *Slc39a11* expression in the respective tissues was confirmed using RT-qPCR (Fig. S4D and E). We then measured serum and tissue Mn concentrations in LKO and IKO mice using ICP-MS. We found that similar to the global *Slc39a11*^−/−^ mice, both male and female LKO mice had significantly lower serum Mn levels compared to sex-matched controls (Fig. 7A); however, with the sole exception of a slight but significant increase in cardiac Mn levels in the female LKO mice, we found no difference in Mn levels between LKO mice and their sex-matched controls in any tissues studied (Fig. 7B and C). In contrast, Mn levels were higher in the serum, liver, and small intestine of female—but not male—IKO mice compared to sex-matched controls (Fig. 7D to F), albeit to a lesser degree than in *Slc39a11*^−/−^ and LKO mice.

### Knocking down *SLC39A11* and exposure to high extracellular Mn increase senescence in cultured human fibroblasts

To examine the role of SLC39A11 in cellular aging, we first generated a line of BJ cells (a human fibroblast cell line) with stable *SLC39A11* knockdown using lentiviral short hairpin RNA (shRNA) expression; knockdown was confirmed using RT-qPCR (Fig. 7G). We then measured senescence-associated β-galactosidase (SA-β-Gal) activity under various conditions to quantify cellular senescence. We found that compared to control cells, sh*SLC39A11*-expressing cells had significantly higher levels of senescence (Fig. 7H and I), suggesting that SLC39A11 may protect against cellular senescence. *SLC39A11* expression in cultured cells was previously reported to respond to extracellular Zn and Mn [37]; moreover, as shown above, we found that SLC39A11 is conserved in vertebrates and serves as a Mn transporter. We therefore examined the effects of Mn and Zn on cellular senescence by exposing the cells to extracellular Mn and Zn, respectively. We found that exposure to Mn—but not Zn—significantly increased cellular senescence to the same extent as knocking down *SLC39A11* (Fig. 7H and I). Thus, we conclude that SLC39A11 may play a major role in protecting against cellular senescence primarily by regulating Mn homeostasis.

## Discussion

Given the rapid increase in our elderly population, scientists have long sought to understand the biology of aging and longevity. Aging is coupled with progressive changes in intercellular communication and dysregulated nutrient homeostasis [38], and characterizing aging-associated genes and biomarkers offers an excellent opportunity to potentially increase longevity and prolong healthy aging [39]. We previously reported that certain SNPs in the *SLC39A11* gene are associated with longevity in male centenarians [20]. However, which ion is transported by SLC39A11, and how this process affects the aging process and longevity, have remained unclear. Here, we functionally characterized SLC39A11 and found that it serves as a conserved Mn transporter in vertebrates, making SLC39A11 the fourth Mn transporter identified after SLC30A10, SLC39A14, and SLC39A8 in vivo.

Our findings reveal several unique features by which SLC39A11 regulates Mn homeostasis. First, SLC39A11 modulates Mn in a relatively subtle manner, as global loss of SLC39A11 results in a relatively small increase in systemic Mn concentration, particularly compared to the considerably larger changes in serum Mn reported in the absence of SLC30A10, SLC39A14, and SLC39A8 [4–8]. Second, we found no apparent accumulation of Mn—which has been shown to cause parkinsonism symptoms—in the brain in *Slc39a11* knockout animals, as reported previously for *Slc30a10* and *Slc39a14* knockout animals [11,12,15]. Third, despite certain similarities between SLC39A11 and SLC39A14—for example, both belong to the SLC39A family of transporters [40] and both are highly expressed in the digestive system [1,21]—hepatocyte-specific *Slc39a14* knockout mice were not reported to develop systemic Mn accumulation [11,13]; in contrast, serum Mn levels increased in our hepatocyte-specific *Slc39a11* knockout mice to the same degree as global *Slc39a11* knockout mice, suggesting that hepatic Slc39a11 plays a key functional role in modulating Mn metabolism.

Interestingly, we found high serum Mn levels in female IKO mice, but not in male IKO mice, suggesting that SLC39A11 plays a sex-specific role in regulating intestinal Mn. An unexpected finding was that Mn concentrations are increased in both the liver and intestine of female IKO mice, suggesting a feedback process for Mn uptake in the intestine. Although this finding is consistent with a previous study in which women were found to be more sensitive than men to Mn absorption [41], the underlying mechanism remains unclear and warrants further study.

As an essential trace element, Mn has been closely linked to human longevity and aging [42]. In addition, elderly men generally have lower serum Mn levels compared to elderly women [43]. Moreover, Guan et al. found a positive association between serum Mn content and the so-called “anti-aging protein” Klotho, but this association was significant only in women [44]. Although these studies suggest gender differences with respect to how Mn metabolism can regulate aging and longevity, the underlying pathways are currently unknown.

Using cultured human fibroblasts, we found that knocking down *SLC39A11* expression and exposure to high extracellular Mn levels increased cellular senescence compared to control cells. Moreover, we found that *slc39a11* mutant zebrafish have higher systemic Mn concentrations and a shorter average lifespan compared to WT controls. In addition, the mutant zebrafish develop an aging-related phenotype, with several potential key implications. First, *slc39a11* mutant zebrafish develop muscular atrophy and have impaired muscle regeneration, providing the first evidence that muscle function is correlated with systemic Mn accumulation due to reduced SLC39A11 activity. This finding may have clinical relevance, as a recent study found that exposure to environmental Mn is a contributing factor in the development of sarcopenia [45], an aging-related condition characterized by progressive loss of muscle mass and strength. This previous report is consistent with the muscular atrophy that we observed in *slc39a11* mutant zebrafish, suggesting that systemic Mn accumulation may serve to trigger muscle aging and aging-related pathological changes. Second, compared to age-matched mutant females, the foregut and midgut of mutant males had more severe damage, suggesting that the Slc39a11 protein may help protect against aging in males, thereby providing a possible mechanism underlying the observed gender differences in the aging-related effects of altering Mn metabolism. Interestingly, a previous meta-analysis found that the rs7210086 SNP in *SLC39A11* was associated specifically with ulcerative colitis [46], which supports the notion that the SLC39A11 protein plays a functional role in the intestine. Future studies are needed to identify the precise mechanisms underlying the function of SLC39A11 in aging and longevity.

In conclusion, we identified SLC39A11 as a conserved Mn transporter and functionally characterized its gender-specific role in regulating aging and longevity. These findings suggest that SLC39A11 may serve as a potential target for preventing aging-related diseases—particularly in men—and provide compelling evidence for examining the precise mechanisms that underlie the association between Mn metabolism and the aging process, which in turn affects longevity.

## Materials and Methods

### Zebrafish and mice

Zebrafish were raised and maintained in accordance with guidelines established by the public technical platform of Zhejiang University School of Medicine. All mice were housed under ventilated, specific pathogen-free conditions under a 12-h/12-h light–dark cycle at 24°C. The mice used in these experiments were on the C57BL/6 background. Global *Slc39a11* (*Slc39a11*^−/−^), LKO, and IKO mice were generated by crossing *Slc39a11^fl/fl^* mice with Dppa3-Cre, Alb-Cre mice, and Villin-Cre mice, respectively (Shanghai Biomodel Organisms). All animal studies were performed in accordance with the guidelines established by the Institutional Animal Use Committee and the Animal Experimentation Ethics Committee of Zhejiang University.

### Generation of *slc39a11* mutant zebrafish using CRISPR/Cas9

The *slc39a11* target sequences were designed using CHOPCHOP (https://chopchop.cbu.uib.no/). The guide RNA (gRNA) template was amplified from the pMD-gata5-gRNA scaffold vector, and in vitro transcription was performed using 1 μg of template DNA and T7 RNA polymerase. The synthesized gRNA was then mixed with Cas9 protein (CP02, PNA Bio Inc.) for microinjection into one-cell-stage embryos. The efficiency of genetically disrupting the target sequence was determined using DNA sequencing and RT-qPCR.

### Zebrafish behavioral experiments

A digital video tracking system (Noldus) running the EthoVision XT 15 software program was used to track the movement and position of WT and *slc39a11* mutant zebrafish. These experiments were performed in a temperature-controlled room (28°C). The fish were first allowed to acclimate to the system for 2 min, followed by data acquisition for 8 min. For the new tank test, each adult fish was placed in a 20 cm × 15 cm tank containing 1 l of water, and the total distance traveled and average swimming speed were recorded. For the open-field test, each adult fish was placed in a 20-cm diameter circle tank containing 1 l of water, and the duration of time the fish was in the center of the tank was recorded. For the dark–light tank test, each adult fish was placed in a 20 cm × 10 cm tank containing 1 l of water, and the duration of time the fish was in the dark area of the tank was recorded.

### AFOG staining

Paraffin-embedded sections were fixed in 10% formalin for 15 min and then washed in phosphate-buffered saline (PBS) containing 0.3% Triton-X for 10 min. Next, the slides were incubated in preheated Bouin’s fixative (AG2331, Acmec), first for 2.5 h at 56°C and then for 1 h at room temperature. The slides were then rinsed for 20 min in tap water and then transferred to 1% phosphomolybdic acid for 5 min. The slides were rinsed with distilled water and then incubated for 4 min with AFOG solution (3 g of acid fuchsin, 2 g of orange G, and 1 g of aniline blue dissolved in 200 ml of acidified distilled water, pH 1.1), after which the slides were rinsed in distilled water to remove excess stain. Finally, the slides were dehydrated in a graded series of ethanol, dipped in xylol, and mounted with neutral balsam. Images were obtained using a Nikon Eclipse Ni microscope.

### Frozen sections

The zebrafish were sacrificed on ice, and the caudal section was excised using surgical scissors, fixed with 4% paraformaldehyde overnight, washed with PBS, and then incubated overnight in a 30% (w/v) sucrose solution. The samples were then transferred to OCT compound (Tissue-Tek) and snap-frozen at −80°C, and 10-μm cryosections were cut using a CM1950 cryostat (Leica).

### Immunofluorescence

Immunofluorescence was performed as described previously [12]. For γ-H2AX staining, a phospho-histone H2AX (Ser^139^) rabbit polyclonal antibody (AF5836, Beyotime) was used. For 4-HNE staining, an anti-4-HNE antibody (ab46545, Abcam) was used. A goat anti-rabbit Alexa Fluor 488 (A0423, Beyotime) was used as the secondary antibody.

### RNA-seq analysis

For RNA-seq analysis, we used the sequencing and bioinformatics analysis services available from LC-Bio Technology Co. Ltd. (https://www.omicstudio.cn).

### Genotyping

Zebrafish and mouse tail samples were obtained, and genomic DNA was extracted using a DNA extraction kit (DR0301250, Easy-Do Biotechnology) in accordance with the manufacturer’s instructions. PCR amplification was then performed using a Taq Plus Master Mix (P212/P213, Vazyme). Zebrafish genotyping was performed using DNA sequencing, while mouse genotyping was performed by electrophoresis through a 2% (w/v) agarose gel.

### Inductively coupled plasma mass spectrometry

The zebrafish embryos and adults were sacrificed by freezing on ice. The mice were anesthetized with pentobarbital sodium and sacrificed by cardiac puncture and exsanguination. All tissues were rapidly excised and weighed, and then frozen in liquid nitrogen or stored at −80°C. Zebrafish samples and mouse tissues weighing >100 mg were added to 1 ml of HNO_3_ (84378, Merk) and digested in a graphite digester at 110°C for approximately 30 min until the tissues were completely digested. For serum samples, 120 μl of serum was added to 200 μl of HNO_3_ and heated in a 95°C water bath for approximately 30 min until the solution was clarified. Next, double-distilled water was added to the digested tissue samples and clarified serum samples to a final volume of 5 ml and 2 ml, respectively. All samples were then filtered through a 0.22-μm cell filter, and various metal ions in the solution were detected using a NexION 300X inductively coupled plasma mass spectrometer (PerkinElmer).

### RNA extraction and RT-qPCR

Total RNA was extracted using the RNA-Quick Purification Kit (RN00, Esunbio). cDNA was synthesized using 2 μg of total RNA as the template, oligo-dT, and SuperScript III Reverse Transcriptase (RR037A, Takara). The cDNA samples were diluted 50-fold and used as the template for RT-qPCR using the Bio-Rad CFX Manager with qPCR SYBR Green Master mix (1202ES03, Yeasen); the primers used are listed in Table S1.

### Cell culture and treatment

BJ cells were cultured in minimum essential medium (MEM) supplemented with 10% fetal bovine serum (FBS; Gibco), 1% sodium pyruvate, and 1% nonessential amino acids (Invitrogen). The cells were transduced with lentivirus expressing shRNA against *SLC39A11* (sh*SLC39A11*) or empty vector (as a negative control) in order to obtain stable *SLC39A11* knockdown cells; transduced cells were selected by culturing in puromycin (1 μg/ml) for 7 days. The cells were then cultured for an additional 14 days in control medium or medium supplemented with either 150 μM MnCl_2_ or 150 μM ZnSO_4_. Finally, SA-β-Gal activity was measured using the Senescence β-galactosidase Staining Kit (C0602, Beyotime) in accordance with the manufacturer’s instructions.

### Mice behavioral experiments

The mice were placed in the center of a 40 cm × 40 cm white box with 40-cm-high walls and allowed to explore freely for 15 min. The light intensity was 290 lx in the center of the arena. Behavioral experiments were digitally recorded and analyzed with a video-imaging system (smart video tracking software, Panlab).

### Statistical analysis

For all experimental data, calculations were performed using Prism (GraphPad). The survival curves were analyzed using the log-rank test. The Gaussian distribution analysis was performed using OriginPro software. An unpaired, 2-tailed Student’s *t* test was used to compare 2 groups, while a one-way analysis of variance (ANOVA) was used to 3 or more groups, and differences were considered significant at *P* < 0.05. Except where indicated otherwise, summary data are presented as the mean ± standard error of the mean (SEM).

## Data Availability

Data are available upon request from F.W. (fwang@zju.edu.cn).
