# Supplementary material for: A Novel Role for the Longevity-Associated Protein SLC39A11 as a Manganese Transporter
Source: Research (Wash D C). 2024 Aug 7;7:0440. doi: 10.34133/research.0440 (PMC11304475; doi:10.34133/research.0440)
Supplement: Supplementary 1 — Figs. S1 to S6 Table S1 [file research.0440.f1.zip › supp Figure S1.pdf]

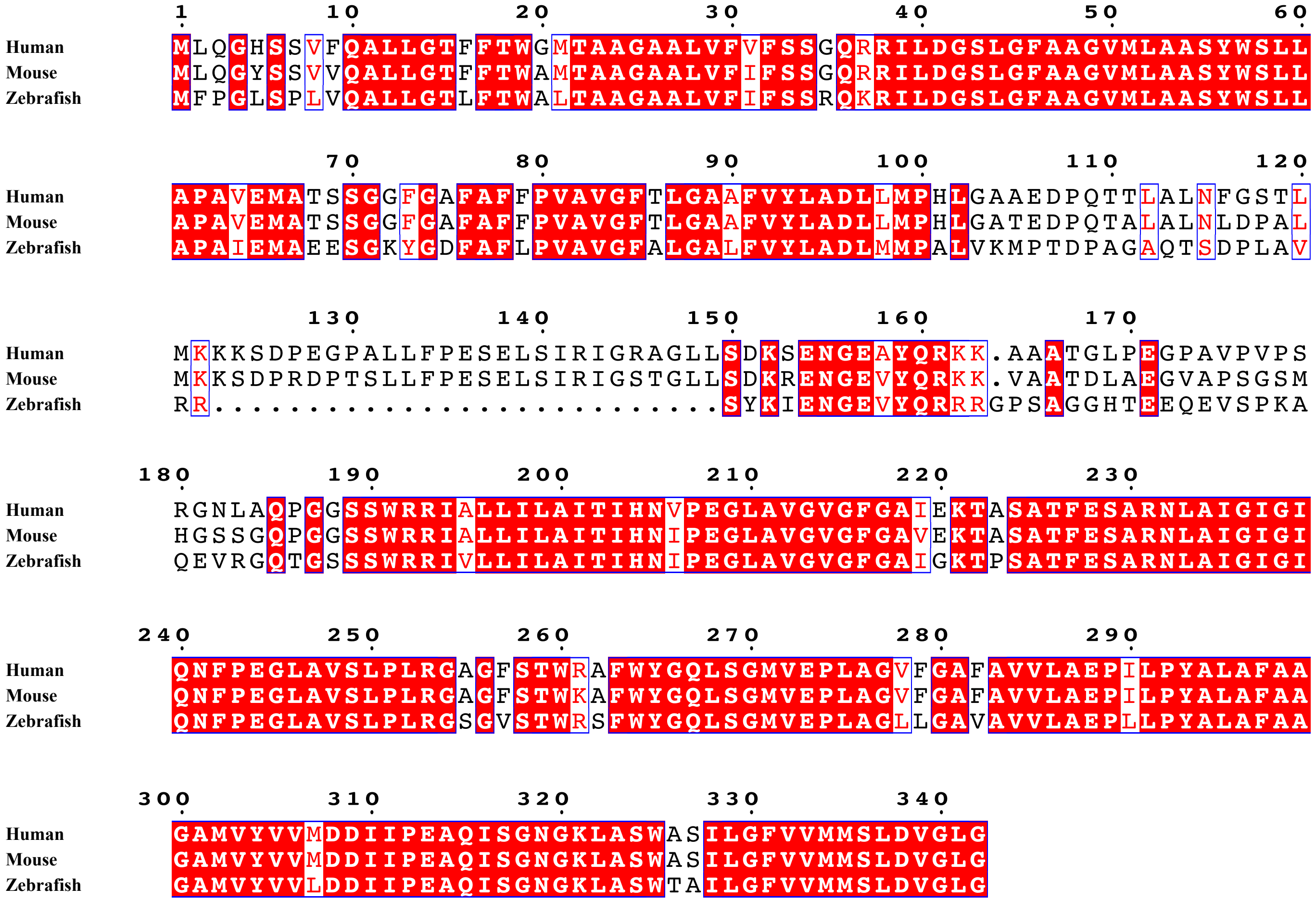
1 10 20 30 40 50 60
Human MLQGHSSVFQALLGTFFTWGMTAAGAALVFVFSSGQRRILDGSLGFAAGVMLAASYWSLL
Mouse MLQGYSSVVQALLGTFFTWAMTAAGAALVFIFSSGQRRILDGSLGFAAGVMLAASYWSLL
Zebrafish MFPGLSPLVQALLGTLFTWALTAAGAALVFIFSSRQKRILDGSLGFAAGVMLAASYWSLL
70 80 90 100 110 120
Human APAVEMATSSGGFGAFAFFPVAVGFTLGAAFVYLADLLMPHLGAAEDPQTTLALNFGSTL
Mouse APAVEMATSSGGFGAFAFFPVAVGFTLGAAFVYLADLLMPHLGATEDPQTALALNLDPAL
Zebrafish APAIEMAEESGKYGDFAFLPVAVGFALGALFVYLADLMMPALVKMPTDPAGAQTSDPLAV
130 140 150 160 170
Human MKKKSDPEGPALLFPESELSIRIGRAGLLSDKSENGEAYQRKK.AAATGLPEGPAVPVPS
Mouse MKKKSDPRDPTSLLFPESELSIRIGSTGLLSDKRENGEVYQRKK.VAATDLAEGVAPSGSM
Zebrafish RR...........................SYKIENGEVYQRRRGPSAGGHTEEQEVSPKA
180 190 200 210 220 230
Human RGNLAQPGGSSWRRIALLILAITIHNVPEGLAVGVGFGAIEKTASATFESARNLAIGIGI
Mouse HGSSGQPGGSSWRRIALLILAITIHNIPEGLAVGVGFGAVEKTASATFESARNLAIGIGI
Zebrafish QEVRGQTGSSSWRRIVLLILAITIHNIPEGLAVGVGFGAIGKTPSATFESARNLAIGIGI
240 250 260 270 280 290
Human QNFPEGLAVSLPLRGAGFSTWRAFWYGQLSGMVEPLAGVFGAFAVVLAEPILPYALAFAA
Mouse QNFPEGLAVSLPLRGAGFSTWKAFWYGQLSGMVEPLAGVFGAFAVVLAEPILPYALAFAA
Zebrafish QNFPEGLAVSLPLRGSGVSTWRSFWYGQLSGMVEPLAGLLGAVAVVLAEPLLPYALAFAA
300 310 320 330 340
Human GAMVYVVMDDIIPEAQISGNGKLASWASILGFVVMMSLDVGLG
Mouse GAMVYVVMDDIIPEAQISGNGKLASWASILGFVVMMSLDVGLG
Zebrafish GAMVYVVLDDIIPEAQISGNGKLASWTAILGFVVMMSLDVGLG
