# Supplementary material for: A Novel Role for the Longevity-Associated Protein SLC39A11 as a Manganese Transporter
Source: Research (Wash D C). 2024 Aug 7;7:0440. doi: 10.34133/research.0440 (PMC11304475; doi:10.34133/research.0440)
Supplement: Supplementary 1 — Figs. S1 to S6 Table S1 [file research.0440.f1.zip › Supplemental materials.docx]

**Supplemental materials**

**
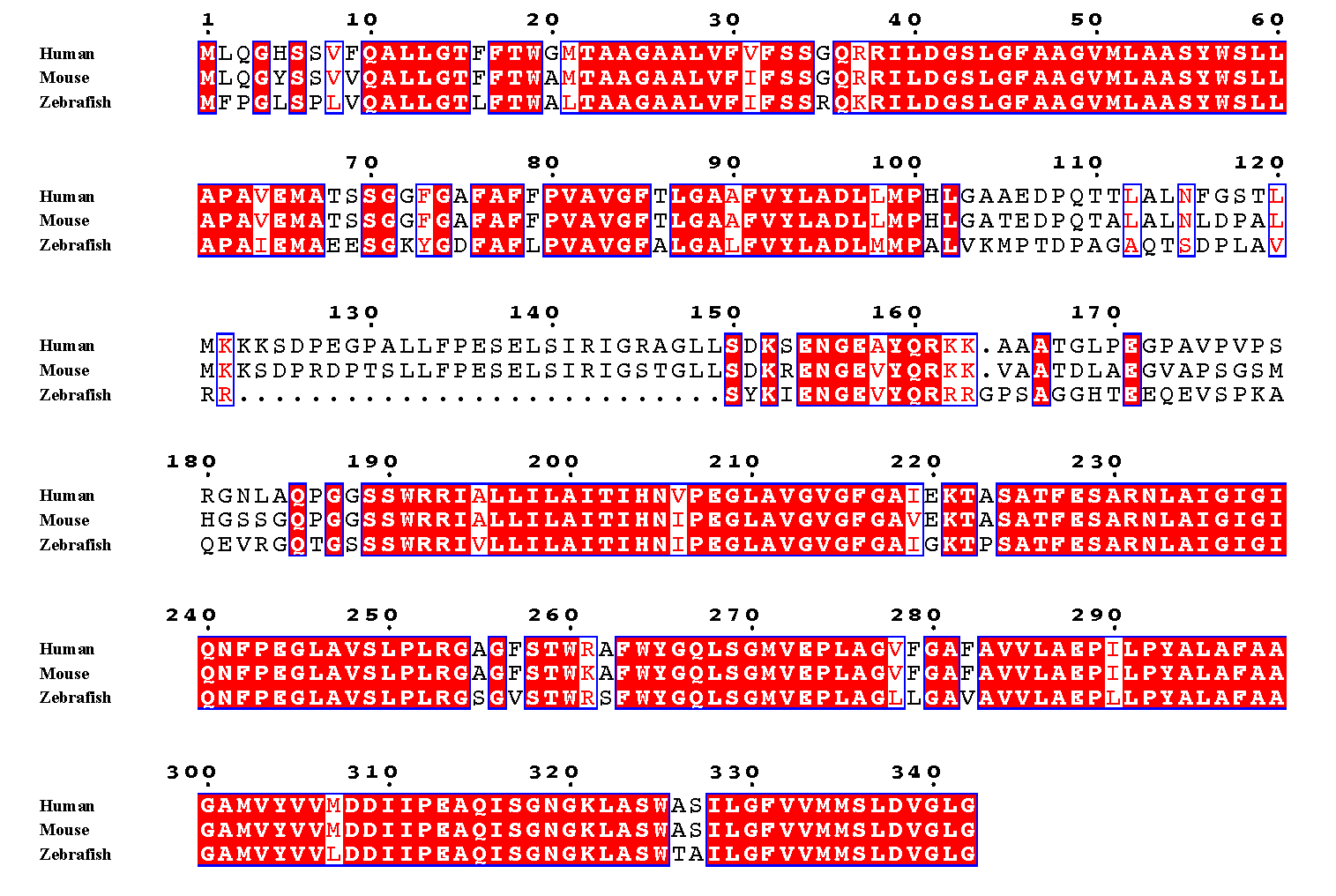
**

**Supplemental Figure S1.** **Protein sequence alignment of SLC39A11 orthologs.** The amino acid sequences of human SLC39A11, mouse Slc39a11, and zebrafish slc39a11 were aligned using ClustalW. Identical residues are written in white with a red background, and semi-conserved residues are written in red in a blue box.

**
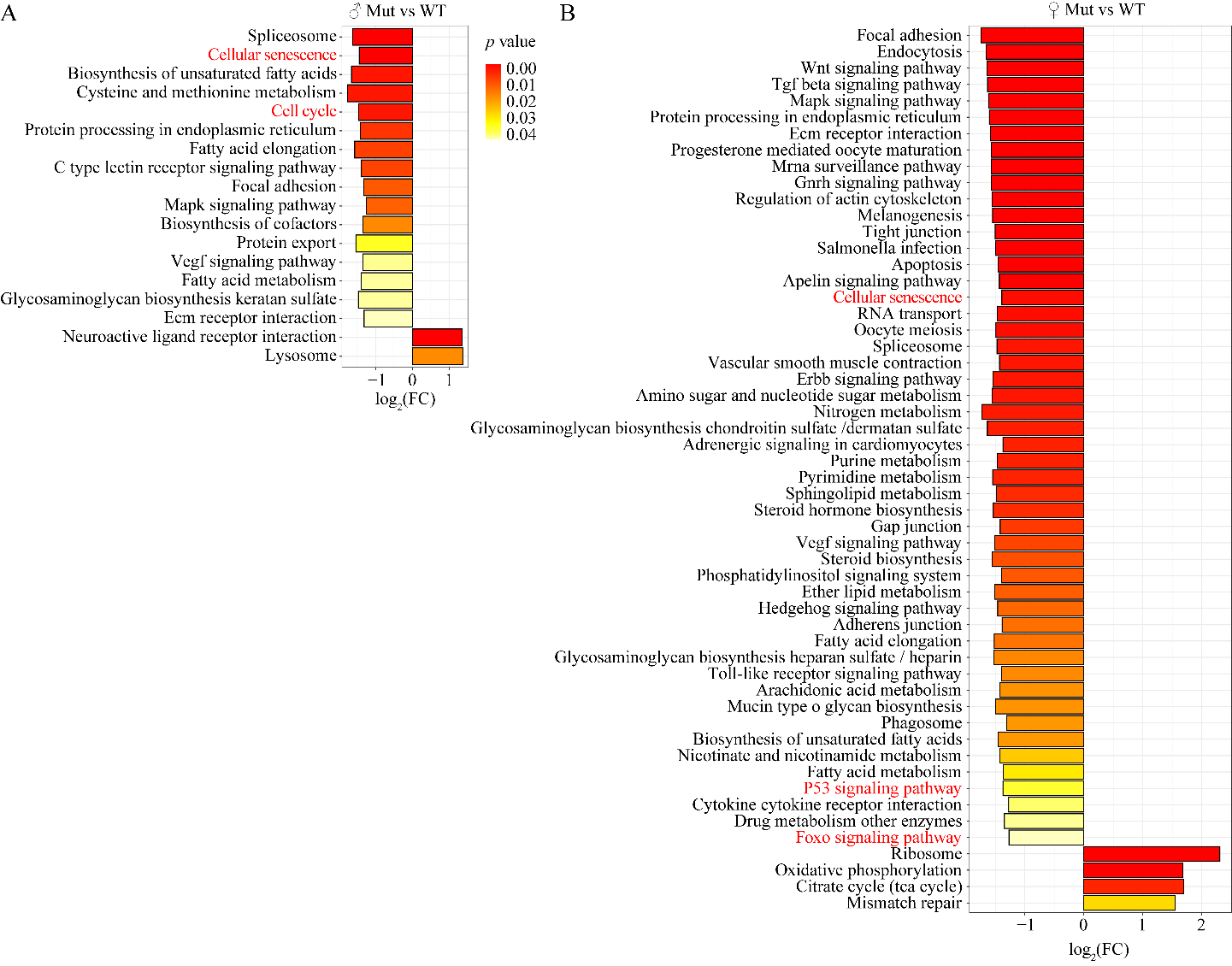
**

**Supplemental Figure S2. RNA-seq analysis reveals significantly altered pathways in muscle samples of WT and *slc39a11* mutant zebrafish. A-B,** Summary of all significant KEGG pathways in male (A) and female (B) *slc39a11* mutants compared to WT controls, based on gene set enrichment analysis (GSEA). Pathways associated with aging are indicated by red dashed boxes.

**
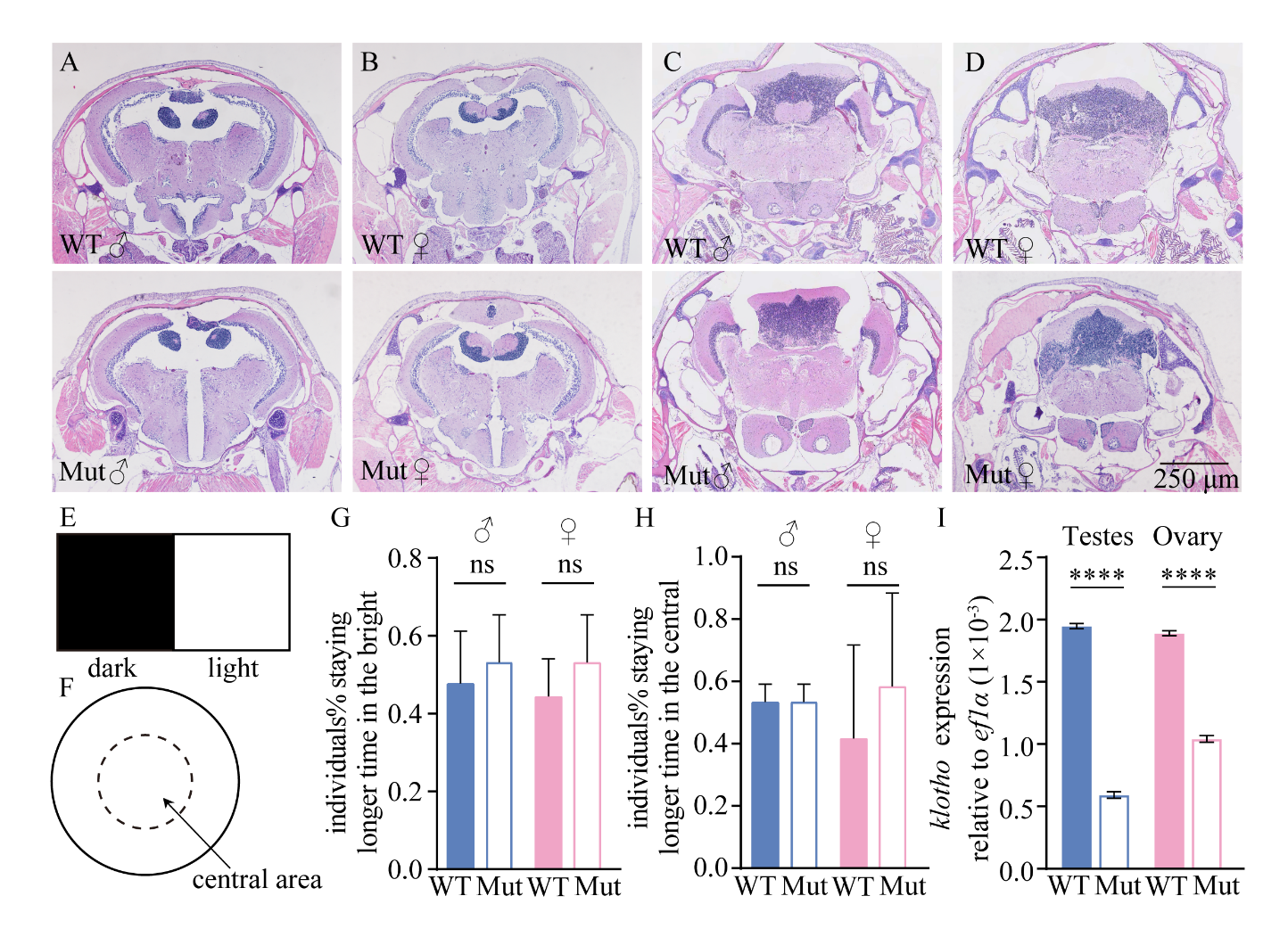
**

**Supplemental Figure S3. Brain morphology, neurological behavior, and *klotho* expression in zebrafish. A-D**, H&E-stained midbrain (A-B) and hindbrain (C-D) sections obtained from male and female WT and Mut zebrafish. **E-F**, Schematic diagrams of the dark-light tank (E) used for the dark-light preference test, and the circular tank (F) using for the open-field test. **G**, summary of the proportion of male and female WT and Mut zebrafish that preferred to stay in the light area of the dark-light tank. **H,** summary of the proportion of male and female WT and Mut zebrafish that preferred to stay in the central area of the circular tank. **I**, summary of *klotho* mRNA measured in the testes and ovaries of WT and Mut zebrafish.

**
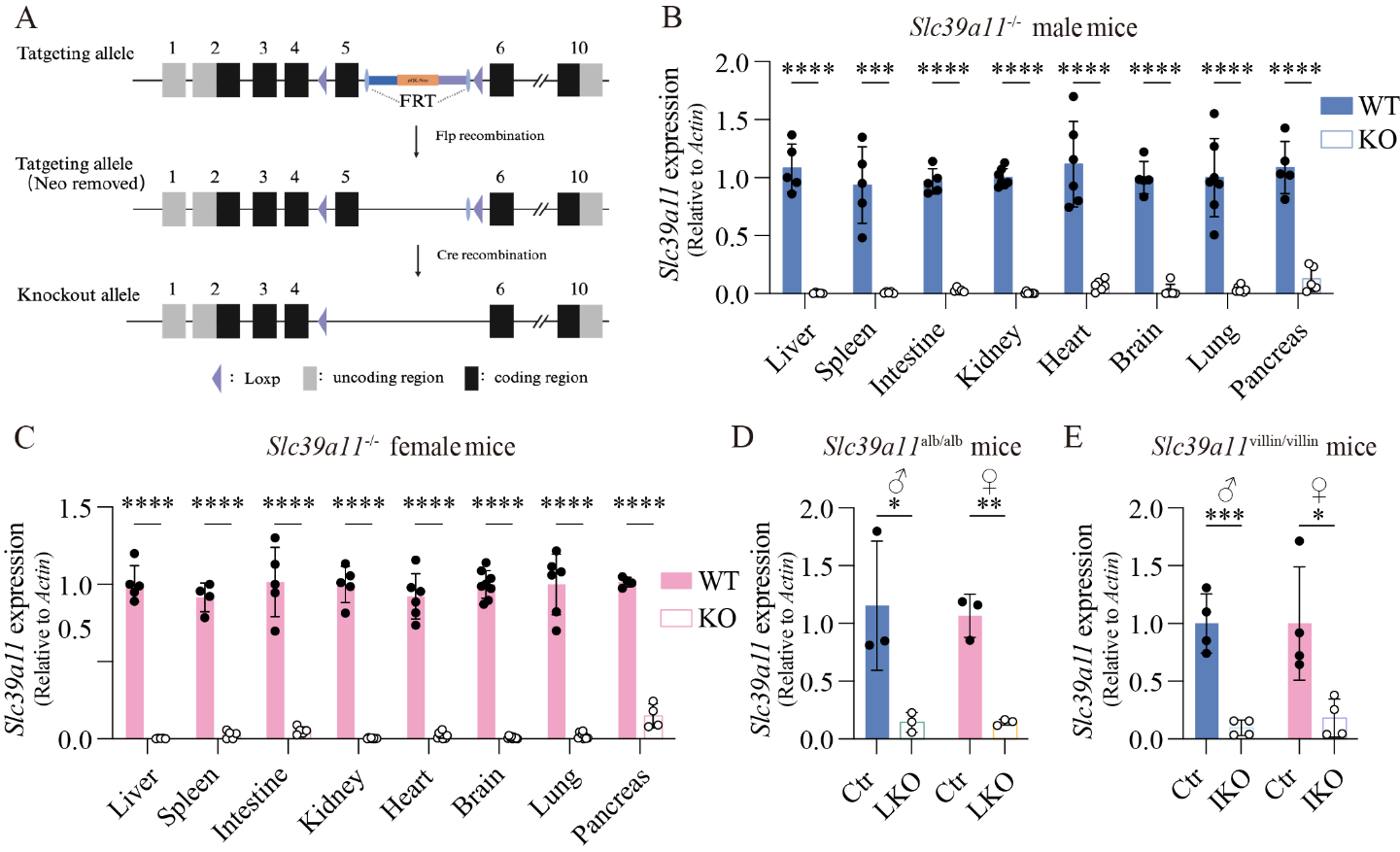
**

**Supplemental Figure S4. Generation and characterization of global (*Slc39a11^-/-^*), liver-specific (LKO), and intestine-specific (IKO) *Slc39a11* knockout mice.**

**A**, strategy for generating *Slc39a11* conditional mice. **B-C,** summary of *Slc39a11* mRNA measured in the indicated tissues of male (B) and female (C) control and global *Slc39a11^-/-^* mice using RT-qPCR. **D-E**, summary of *Slc39a11* mRNA measured in the liver (D) and intestine (E) of male and female control and *Slc39a11^alb/alb^* (LKO, D) and *Slc39a11^vill/vill^* (IKO, E) mice using RT-qPCR. **P*<0.05, ***P*<0.01, ****P*<0.001, and *****P*<0.0001.


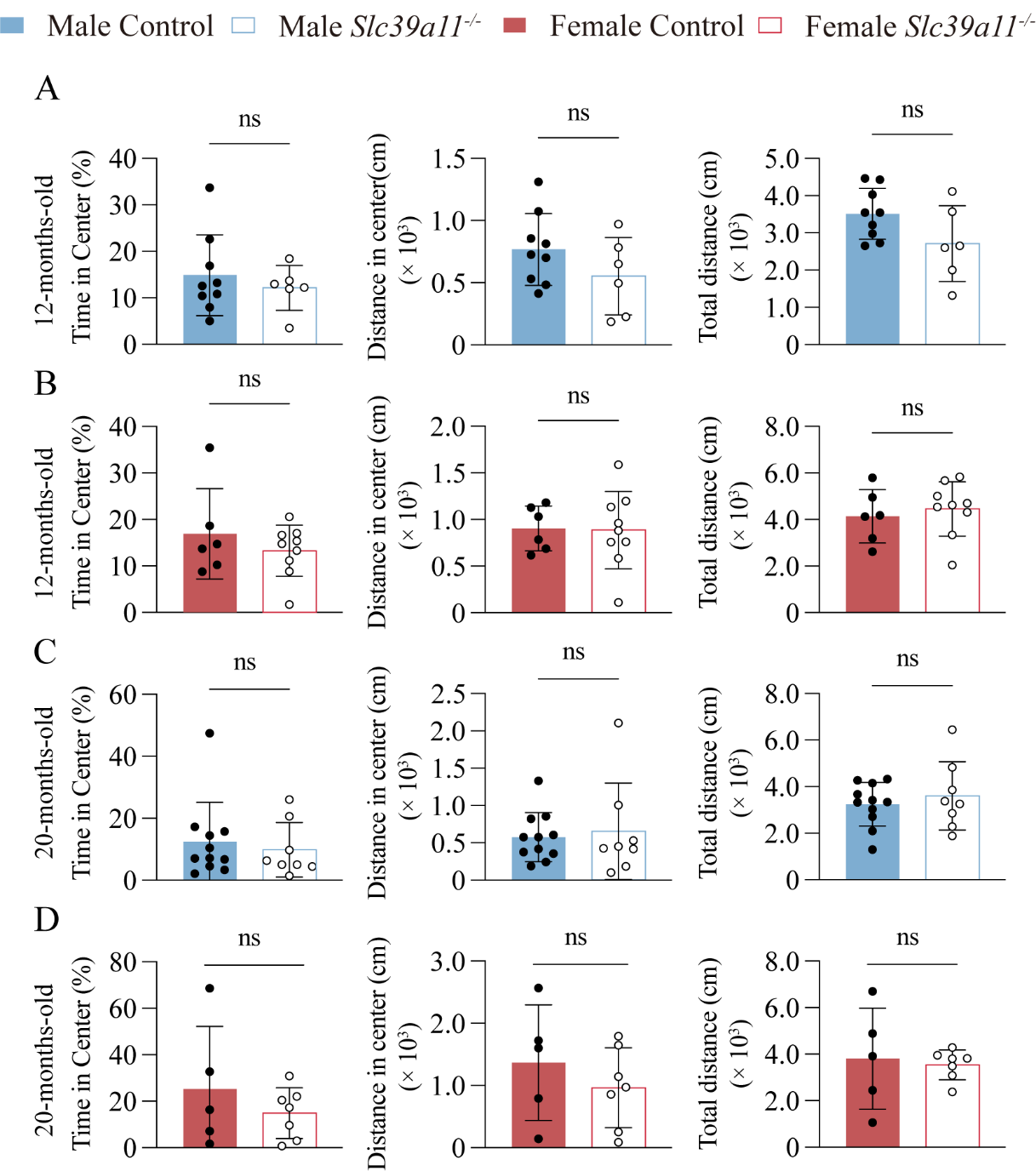


**Supplemental Figure S5. Neural behavior is unaffected in aged *Slc39a11^-/-^* mice.**

**A-D**, summary of the results of the open-field test in 12-month-old (A-B) and 20-month-old (C-D) male (A and C) and female (B and D) control and *Slc39a11^-/-^* mice. The percentage of time spent in the center, the distance traveled in the center, and total distance traveled are shown in the left, middle, and right panels, respectively. ns, not significant.


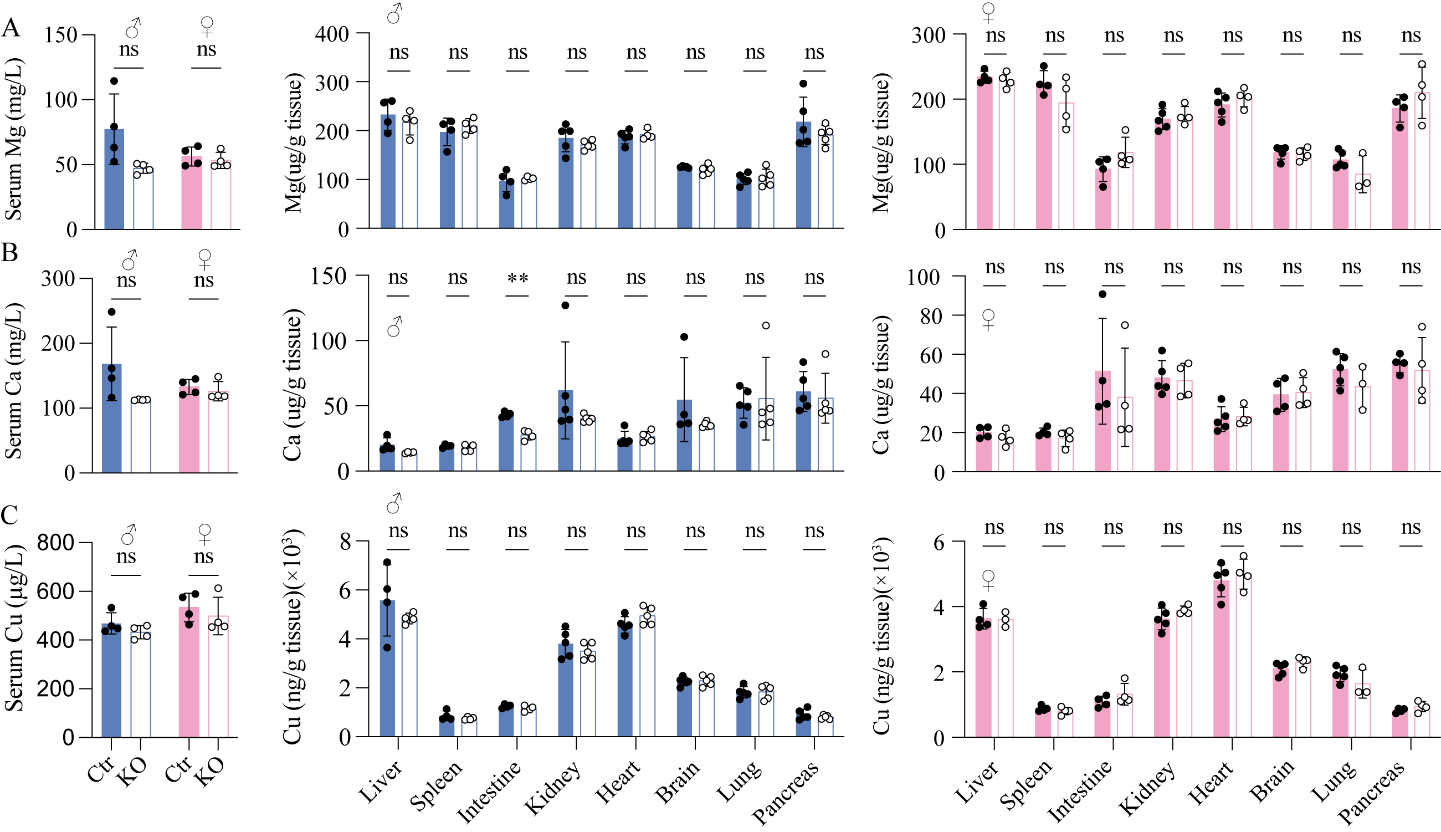


**Supplemental Figure S6. Serum and tissue cation levels in** **control and global *Slc39a11* knockout (KO) mice.**

**A-C**, summary of Mg (A), Ca (B), and Cu (C) concentration measured in the serum and indicated tissues of male and female 2-month-old control and *Slc39a11* KO mice. ***P*<0.01 and ns, not significant.

**Supplementary Table S1. Sequences of the primers used for RT-qPCR analysis**

| Gene | Forward primer (5’ 🡪 3’) | Reverse primer (5’ 🡪 3’) |
| --- | --- | --- |
| Mouse *Slc39a11* | GAGGGCACCCTACAGTATCC | AGGAAGCCGCTAACATGACC |
| Mouse *Actin* | CATTGCTGACAGGATGCAGAAGG | TGCTGGAAGGTGGACAGTGAGG |
| Zebrafish *slc39a11* | TTCTCCAGCAGACAGAAGCG | AACGAACAGAGCTCCCAGTG |
| Zebrafish *ef1a* | CTGGAGGCCAGCTCAAACAT | ATCAAGAAGAGTAGTACCGCTAGCATTAC |
| Zebrafish *klotho* | AATCTGCTGAAGGCTCACGCT | TGTCTGGTTCTGCTGGGTTTG |
| Zebrafish *myod1* | CCCAACGTGTCAGACGAGAA | GGAAATTCGCTCCACGATGC |
| Zebrafish *myog* | GCTCCACATACTGGGGTGTC | GGGGACACAGTGATCAGACG |
| Zebrafish *sod2* | TTCAACCCCCTGTTAGGTGC | ATGTTGCATGGTGCTTGCTG |
| Zebrafish *p62* | GAGCGTCAGTGAGGGAACAA | TCCTGTCGAAGGATCCACCT |
| Human *SLC39A11* | GCTGATGGAAGTGCAGTGGG | TCTAAGATCCGCCTCTGTCCA |
| Human *ACTIN* | CTGAGGAGCACCCTGTGCT | GTTGAAGGTCTCAAACATGATCTG |
