# Supplementary figures and images for: A Novel Role for the Longevity-Associated Protein SLC39A11 as a Manganese Transporter
Source: Research (Wash D C). 2024 Aug 7;7:0440. doi: 10.34133/research.0440 (PMC11304475; doi:10.34133/research.0440)

A

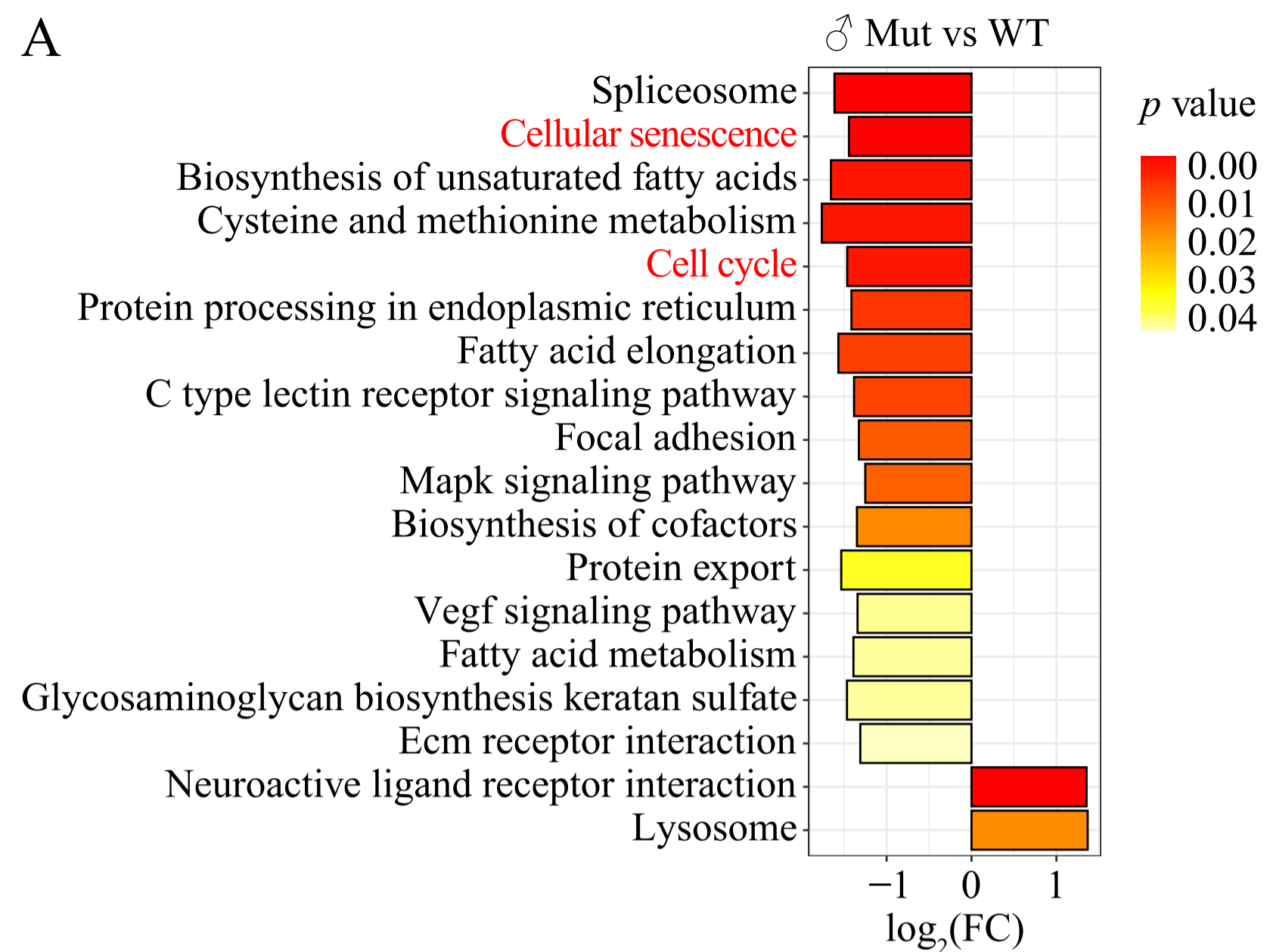

B

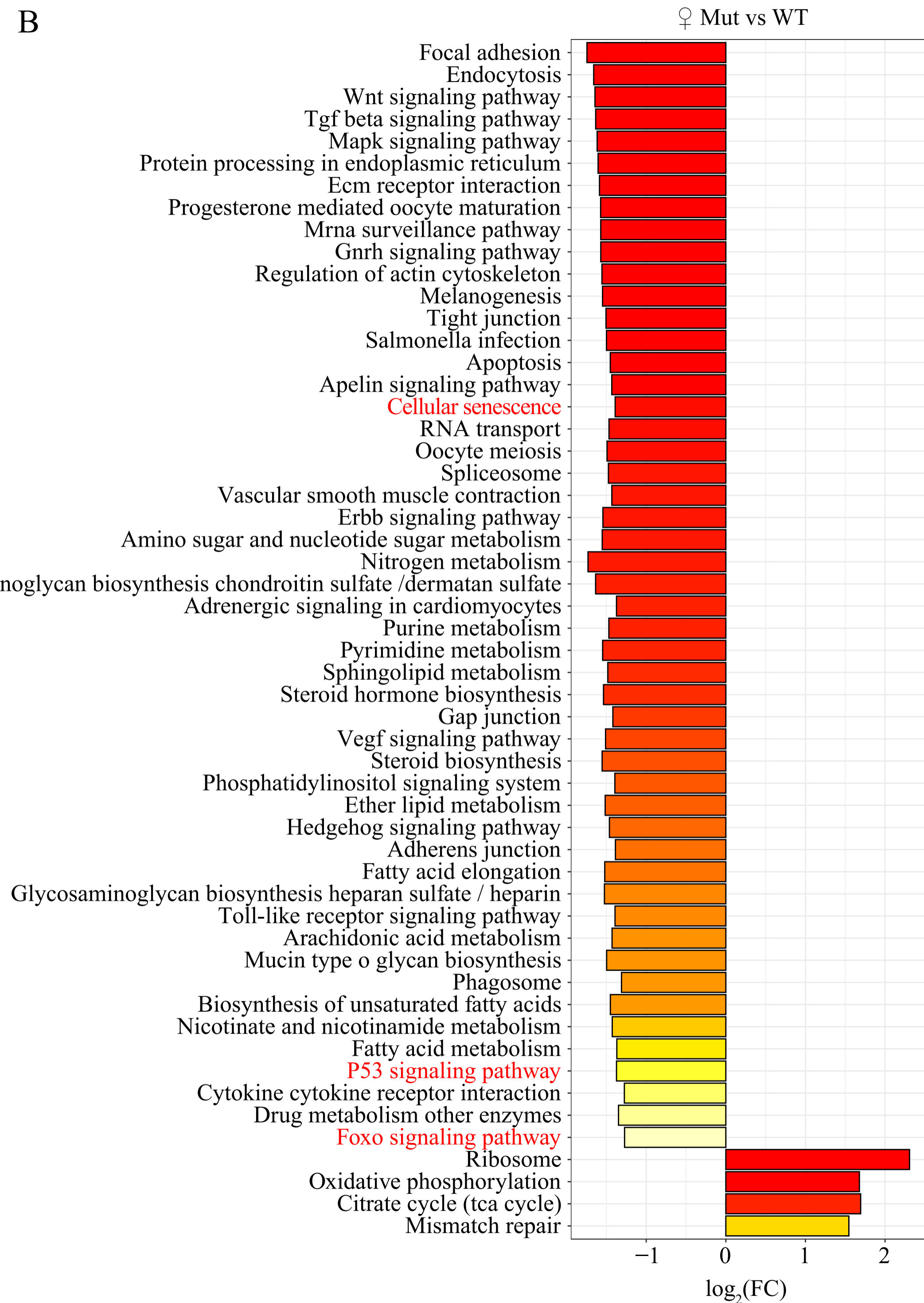

Supplement: Supplementary 1 — Figs. S1 to S6 Table S1 [file research.0440.f1.zip › supp Figure S2.pdf]

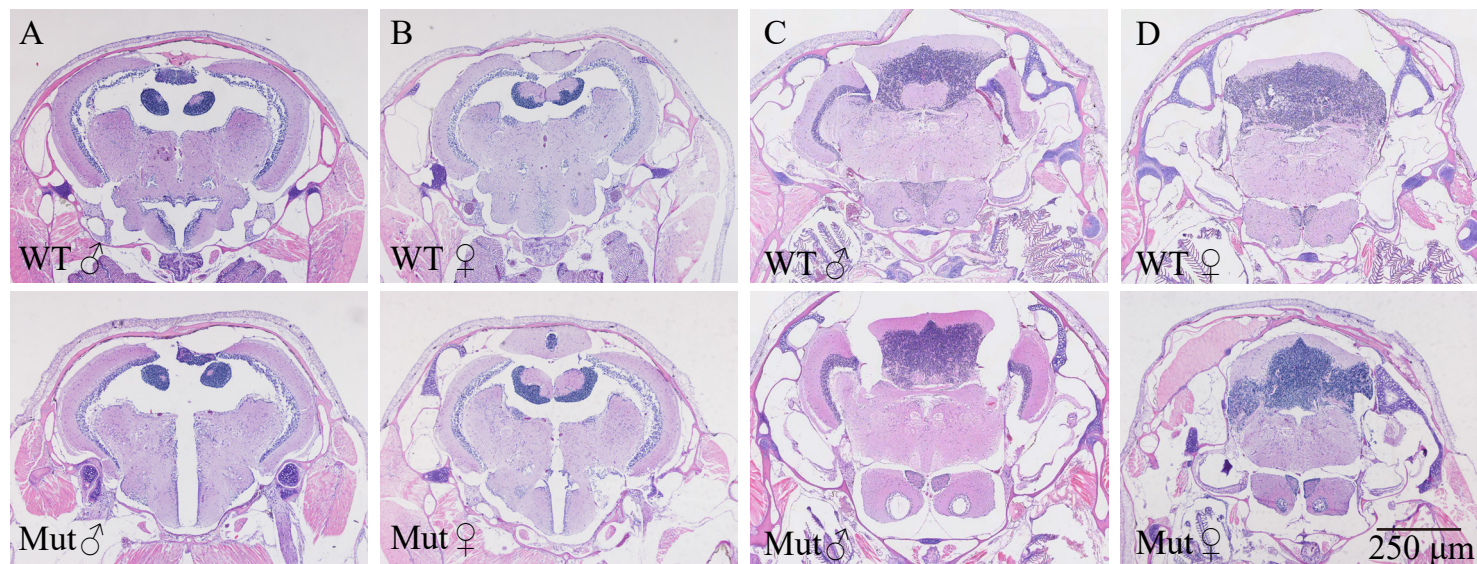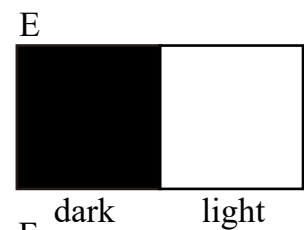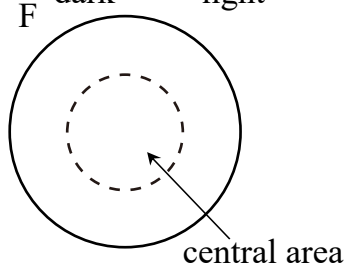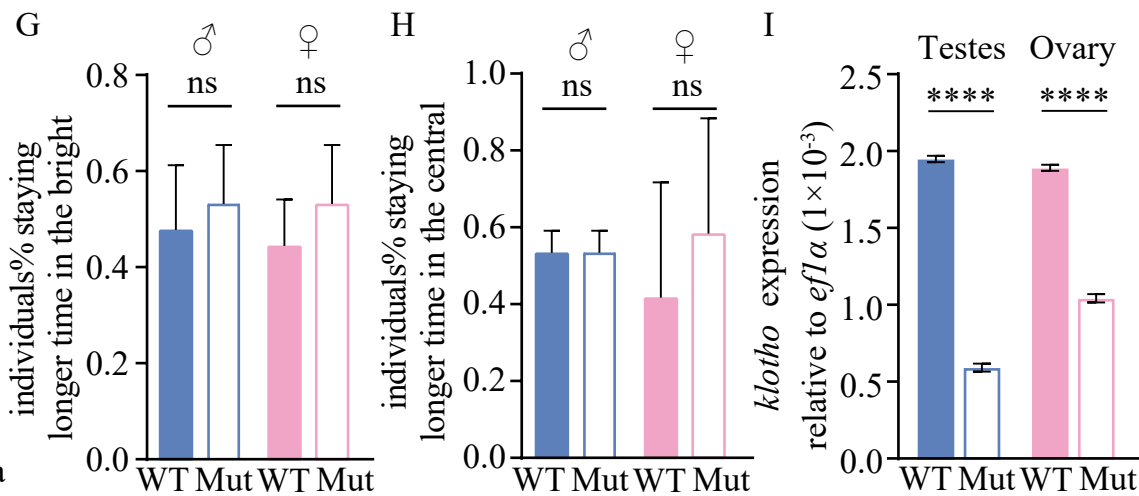

Supplement: Supplementary 1 — Figs. S1 to S6 Table S1 [file research.0440.f1.zip › supp Figure S3.pdf]

A

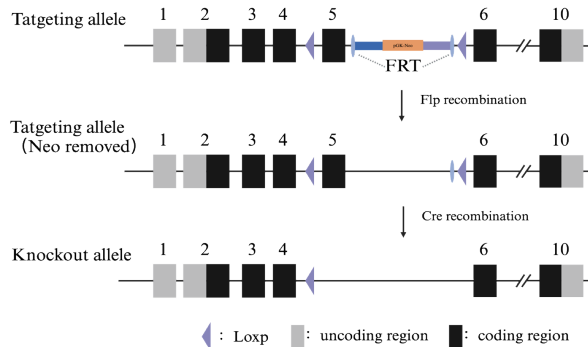

B

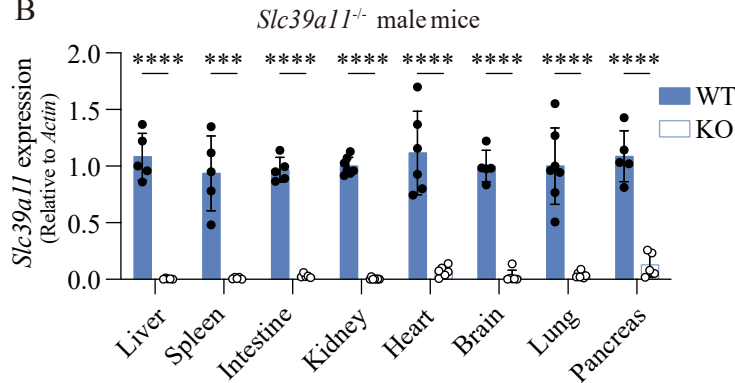

C

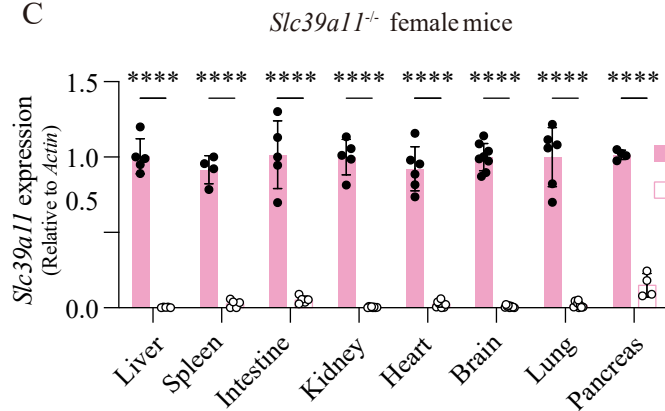

D

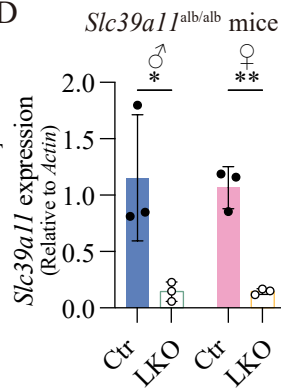

E

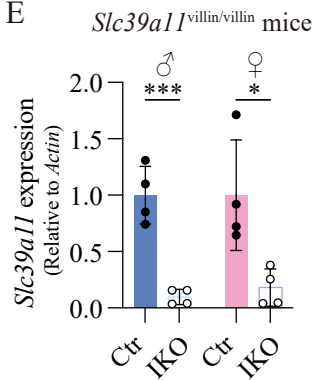

Supplement: Supplementary 1 — Figs. S1 to S6 Table S1 [file research.0440.f1.zip › supp Figure S4.pdf]

■ Male Control □ Male *Slc39a11*<sup>-/-</sup> ■ Female Control □ Female *Slc39a11*<sup>-/-</sup>

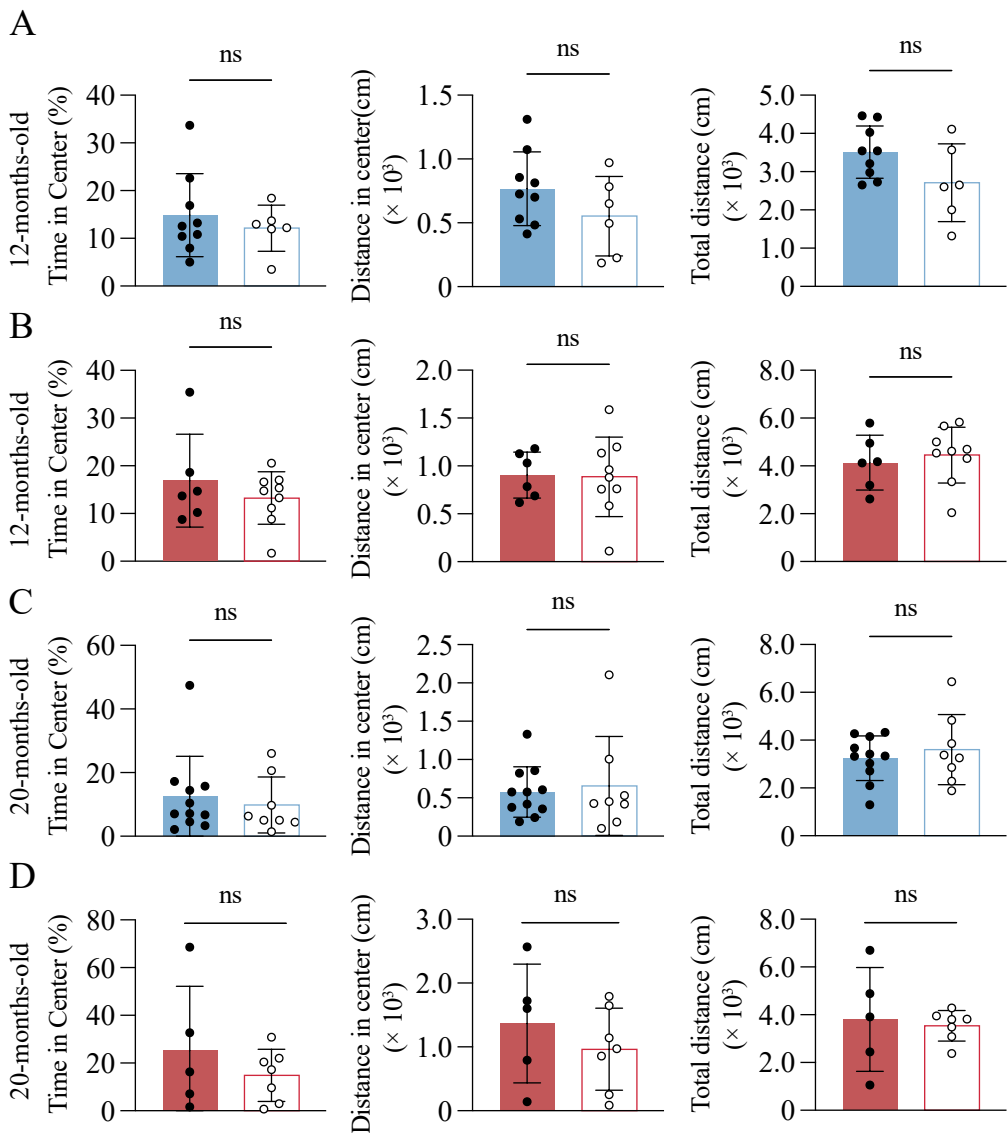

Supplement: Supplementary 1 — Figs. S1 to S6 Table S1 [file research.0440.f1.zip › supp Figure S5.pdf]

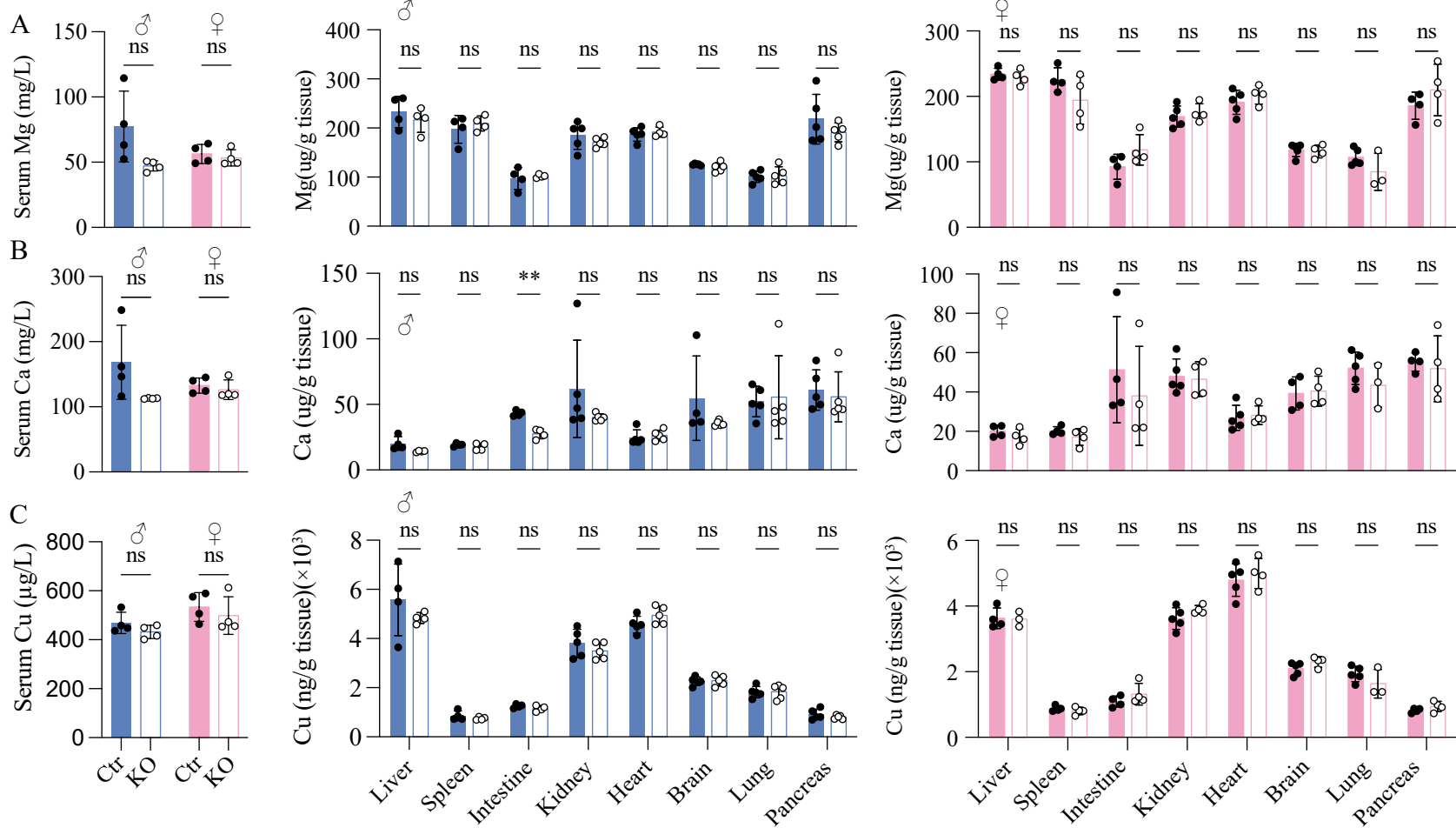

Supplement: Supplementary 1 — Figs. S1 to S6 Table S1 [file research.0440.f1.zip › supp Figure S6.pdf]
